# Identification of Potential Biomarkers of Depression and Network Pharmacology Approach to Investigate the Mechanism of Key Genes and Therapeutic Traditional Chinese Medicine in the Treatment of Depression

**DOI:** 10.1155/2021/2165632

**Published:** 2021-12-31

**Authors:** Yucong Shi, Dan Chen, Shengsuo Ma, Huachong Xu, Li Deng

**Affiliations:** ^1^College of Traditional Chinese Medicine, Jinan University, Guangzhou 510632, China; ^2^Department of Obstetrics and Gynecology, Central Hospital of Wuhan, Affiliated to Huazhong University of Science and Technology, Wuhan 430014, China

## Abstract

**Background:**

To explore the potential target of depression and the mechanism of related traditional Chinese medicine in the treatment of depression.

**Method:**

Differential gene expression in depression patients and controls was analyzed in the GEO database. Key genes for depression were obtained by searching the disease databases. The COREMINE Medical database was used to search for Chinese medicines corresponding to the key genes in the treatment of depression, and the network pharmacological analysis was performed on these Chinese medicines. Then, protein-protein interaction analysis was conducted. Prediction of gene phenotypes was based on Gene Ontology (GO) and Kyoto Encyclopedia of Genes and Genomes (KEGG) pathway enrichment scores.

**Results:**

The total number of differentially expressed genes in the GEO database was 147. Combined with the GEO dataset and disease database, a total of 3533 depression-related genes were analyzed. After screening in COREMINE Medical, it was found that the top 4 traditional Chinese medicines with the highest frequency for depression were *Paeonia lactiflora* Pall., Crocus sativus L., *Bupleurum chinense* DC., and *Cannabis sativa* L. The compound target network consisted of 24 compounds and 138 corresponding targets, and the key targets involved PRKACA, NCOA2, PPARA, and so on. GO and KEGG analysis revealed that the most commonly used Chinese medicine could regulate multiple aspects of depression through these targets, related to metabolism, neuroendocrine function, and neuroimmunity. Prediction and analysis of protein-protein interactions resulted in the selection of nine hub genes (ESR1, HSP90AA1, JUN, MAPK1, MAPK14, MAPK8, RB1, RELA, and TP53). In addition, a total of four ingredients (petunidin, isorhamnetin, quercetin, and luteolin) from this Chinese medicine could act on these hub genes.

**Conclusions:**

Our research revealed the complicated antidepressant mechanism of the most commonly used Chinese medicines and also provided a rational strategy for revealing the complex composition and function of Chinese herbal formulas.

## 1. Introduction

Depression is a prevalent mental disorder ranked as the leading nonfatal cause of disability by the World Health Organization [1]. Major depressive disorder (MDD) is one of the most common psychiatric disorders resulting in a lifetime disability. It is also an important illness associated with suicidal ideation and completed suicide [2–4]. Depression influences various diseases, such as cardiovascular disease and diabetes mellitus [5]. Research on the treatment of depression has been extensive and has shown that depression can be treated with three different forms of psychotherapies: (1) antidepressants and other medications that augment antidepressant action, (2) evidence-based psychotherapy such as cognitive-behavior therapy (CBT) and interpersonal psychotherapy (IPT), and (3) somatic nonpharmacological treatments including electroconvulsive therapy (ECT), repetitive transcranial magnetic stimulation (rTMS), and vagus nerve stimulation (VNS) [6]. However, the treatment effects were limited.

Traditional Chinese medicine (TCM) has been practiced over centuries in Asia and relies on tremendous empirical knowledge [7]. The use of TCM in the treatment of depression has a long history. TCM was found to be superior to antipsychotic drugs in its effects on antianxiety/depression and antipsychomotor inhibition. Traditional Chinese medicine has been proven to have mild antidepressant benefits with few side effects [8]. Xiaoyaosan (XYS) is a traditional Chinese medicine prescription and has been widely used for centuries to treat depressive conditions. XYS was divided into five different polar fractions to explore the antidepressant activity. The results obtained showed that the petroleum ether fraction of XYS is the most effective fraction, suggesting that lipophilic components probably contribute to the antidepressant effect of XYS [9]. Gao et al. [10] showed that the petroleum ether fraction of Bupleuri Radix (PBR) produces an antidepressant effect by regulating glycometabolism, amino acid metabolism, sphingolipid metabolism, glycerophospholipid metabolism, and fatty acid metabolism. Kaixin Jieyu Fang can alleviate cerebral white matter damage, and the underlying mechanism is associated with the regulation of Bcl-2/Bax protein and mRNA expression, which is one of the possible mechanisms behind the protective effect of Kaixin Jieyu Fang against vascular depression [11].

*Paeonia lactiflora* Pall. has analgesic, anti-inflammatory, antioxidant, antidepression, anticardiovascular diseases; antineurodegenerative diseases; and other biological activities [12]. Total glucosides of paeony can also reduce the extent of cerebral infarction by increasing the content of ATP in the hippocampus and enable ischemic site reperfusion to treat depression [13]. *Paeonia lactiflora* Pall. and *Bupleurum chinense* DC. are the basic combination of *Bupleurum* prescriptions such as Xiaoyao powder, Sini powder, and Xiaochaihu decoction. The two drugs together play the function of nourish blood, soften the liver, and have the characteristics of dispersing and collecting, harmonizing qi and blood, and combining dispersing and softening, which accord with the physiological characteristics of the liver of “body Yin and use Yang” [14]. The drug pair of *Paeonia lactiflora* Pall. and *Bupleurum chinense* DC. improved depressive symptoms in rats by reversing the decrease in monoamine neurotransmitters. At the same time, it can enhance the expression of neurotrophic factors, brain-derived neurons, and receptor tyrosine protein kinases in the hippocampus of rats and achieve an antidepression effect [15, 16]. *Paeonia lactiflora* Pall. and *Bupleurum chinense* DC. [17] participate in the Ca^2 +^ signaling pathway to the target of action and thus can inhibit the damage caused by Ca^2 +^ overload. Hosseinzadeh and Noraei [18] studied *Crocus sativus* L. water extract and its effective component (saffron element and saffron formaldehyde) in hypnosis, fight anxiety, autonomic activity, and the action of sport harmonious ability respect through experiment of sodium of pentobarbital, maze, opening experiment, and Rotarod experiment. Emp oil can partially improve the depressive behavior and cognitive ability of mice and reduce the expression of IL-1*β*. The CBD can improve the anxiety symptoms of mice, increase learning and cognitive functions, and inhibit the cortex and hippocampus of brain tissue [19]. Cannabidiol (CBD) is one of the phenols in *Cannabis sativa* L. oil [20]. CBD can quickly cross the blood-brain barrier and can be used as a treatment for nervous system diseases caused by stress [21] and [22]. The results of forced swimming experiments in mice suggest that CBD can reduce the immobilization time in mice and may have antidepressant effects.

The traditional Chinese medicine compound preparation is easy to obtain and rich in various active ingredients. It has the characteristics of acting on multiple targets, and a single component cannot completely reveal its pharmacological activity. In recent years, the application of high-throughput platforms in gene expression has been widely used in clinical studies such as disease prognosis and targeted drug development. Therefore, it is of great significance to explore the key molecules in depression and identify effective Chinese medicine for the treatment of depression. In this study, 15 datasets generated by depression microarray technology and 5 disease gene databases were analyzed to explore the key genes of depression, and the mechanism of related traditional Chinese medicine in the treatment of depression was approached by network pharmacology analysis.

## 2. Method

### 2.1. The Prediction of Known Therapeutic Targets Acting on Depression

The Gene Expression Omnibus (GEO) database [23] (http://www.ncbi.nlm.nih.gov/geo) is the largest, most comprehensive, and publicly available source of gene expression data. We searched all depressive gene expression datasets from the GEO database up to July 2021 using the keywords “depression” and “depressive disorder.” The datasets of nonhuman, nonbrain, noncase-control experiments, and nonmajor depression were removed.

In addition, five disease databases of the DrugBank, GeneCards, OMIM, PharmGkb, and TTD were retrieved. The websites of these five databases are listed in Supplementary Table S1.

### 2.2. Data Processing

Genes collected from the GEO database were analyzed to determine the different genes between the brain tissues of patients with depression and normal people, and then the genes were combined. Finally, we combined the total of differentially expressed genes (DEGs) with the depression-related genes found in the disease database and excluded the duplicates.

### 2.3. Screening of Traditional Chinese Medicine for the Treatment of Depression

COREMINE Medical (https://www.pubgene.com/coremine-medical/) is the world's most advanced medical information retrieval platform jointly developed by Norway, the Chinese Academy of Sciences, the Chinese Academy of Medical Sciences, the US National Library of Medicine, and other institutions. By entering the name of depression disease into this database, potential drugs associated with depression disease can be retrieved. According to the frequency statistics of the screened TCM, the top herbs with the highest frequency were obtained.

### 2.4. Chemical Ingredient Database and Putative Target Building of the Herbs

To determine the chemical ingredients of the four herbs, we performed a search by the Traditional Chinese Medicine Systems Pharmacology Database TCMSP. We type complete Pinyin of each drug in the database and then combine with the existing research results in the literature at the same time, through multiple preexperiments. Finally, we selected oral bioavailability (OB) ≥30% and drug-likeness (DL) ≥0.18 as TCMSP property parameters to predict the effective components in the four herbs.

### 2.5. Construction of Drug-Target-Disease Interaction Network

After obtaining compound-related targets and depression disease targets in the four herbs, the intersection target genes of the two were obtained. The visualization of the drug-target-disease interaction network was constructed with Cytoscape 3.8.0.

### 2.6. Protein-Protein Interaction Analysis

After obtaining the relevant targets of the four herbs and depression disease targets, we took the intersection target genes of the two and constructed the protein interaction network (PPI) online by STRING (http://string-db.org/), which was made to explore the interaction between known and predicted compounds and proteins [24]. Only proteins that interacted directly with each compound in HPXL were selected as the presumed targets. To ensure the reliability of the data, we set the “minimum required interaction score” to communicate the protein-protein interaction information with a combined score ≥0.9.

Then, Cytoscape 3.8.0 was applied to visualize the structure of the protein network. In the process of protein network construction, a force-oriented algorithm was used to distribute nodes reasonably to produce a clear visual effect for protein interaction data (PPI). To use a short sentence for the overview process of how we generated a PPI network graph, we downloaded and saved TSV files from the previous analysis results and then imported them into Cytoscape 3.8.0 software. CytoNCA plug-in was used for topology analysis. We selected core targets whose degree centrality was greater than the average degree value for further analysis. The PPI network diagram of the core targets was constructed using Cytoscape 3.8.0.

### 2.7. Gene Ontology (GO) Analysis

Gene Ontology (GO) analysis [25] was conducted on the intersection of drug targets and disease targets through the clusterProfiler R package. The clusterProfiler R package was utilized to obtain significant enrichment results and figures of GO analysis. In this study, only the top ten most significant GO items were retained.

### 2.8. Molecular Docking

AutoDockTools 1.5.6 software was used to measure semiflexible molecular docking. The compounds' 3D structures were downloaded from the PubChem database as ligands. Core targets were used as target proteins, and proteins were obtained from the Research Collaboratory for Structural Bioinformatics Protein Databank (RCSB PDB) as receptors. Polar hydrogen was added to the treated receptor files using AutoDock computational software.

Next, the parameters of the docking box were set with the AutoGrid tool to find and record the optimal docking position between the receptor and the ligand. If analysis of the affinity (kJ·mol^−1^) of molecular docking indicated binding energy less than 0, we assumed that the ligand and receptor could spontaneously bind. In this study, affinity ≤ −5.0 kJ·mol^−1^ was selected as the screening basis. Finally, PYMOL software was used to analyze and observe the docking results of the compounds and proteins.

**Figure 1:**
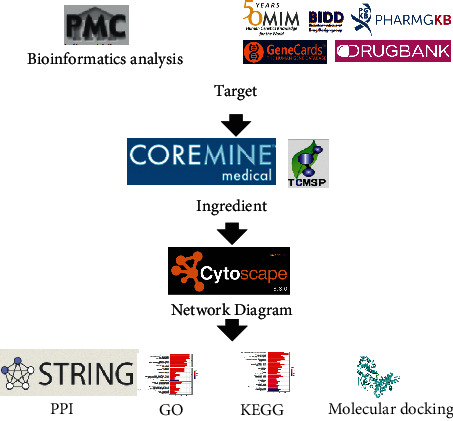
Flowchart of the network pharmacology-based strategy. TCMSP: Traditional Chinese Medicine Systems Pharmacology; GeneCards: retrieve human genetic information; OMIM: Online Mendelian Inheritance in Man; PPI: protein-protein interaction; TTD: Therapeutic Target Database.

The flowchart of strategy based on network pharmacology is shown in Figure 1.

## 3. Results

### 3.1. Disease Target Genes

Thirteen case-control brain gene expression datasets of human major depressive disorder were obtained. GSE53987 in these 13 datasets contains data from the hippocampus (HPC), prefrontal cortex (PFC), and associative striatum (STR), so it is divided into three subdatasets: GSE53987–HPC, GSE53987–STR, and GSE53987–PFC. Therefore, this study contains 15 datasets of severe depression, and the GEO addresses corresponding to these datasets are shown in Table 1.

### 3.2. Prediction of Compounds-Related Targets

**Figure 2:**
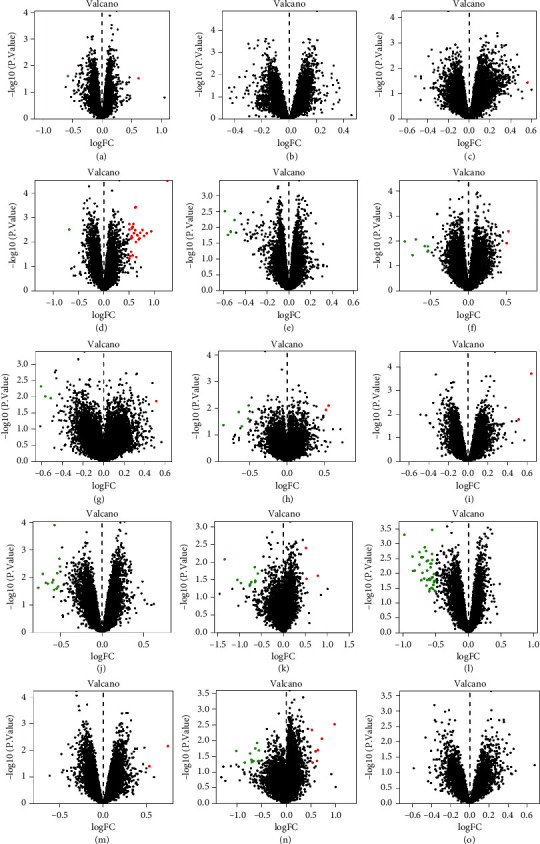
Volcano diagram of differential genes. A: GSE53987-HIP; B: GSE53987-PFC; C: GSE53987-STR; D: GSE54562; E: GSE54563; F: GSE54564; G: GSE54565; H: GSE54566; I: GSE54567; J: GSE54568; K: GSE54570; L: GSE54571; M: GSE54572; N: GSE54575; O: GSE12654. Red dots represent upregulated gene expression and the green dots represent downregulated gene expression.

**Figure 3:**
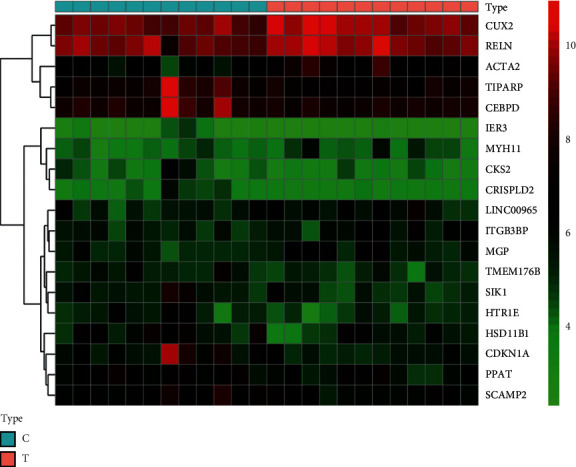
Heat map of GSE54575. C: gene analysis of brain tissue in the normal group. T: analysis of brain tissue genes in the depression group.

After preliminary screening, it was found that, by comparing the gene expression between the depression group and the control group, the total number of differential genes in the GEO database was 147. There were 50 upregulated genes and 89 downregulated genes in 15 GSE datasets. The volcanic map analysis of differential genes in each dataset is shown in Figure 2. The heat map of dataset 14-GSE54575 is shown in Figure 3, where the red represents the upregulated gene expression and the blue represents the downregulated gene expression.

**Figure 4:**
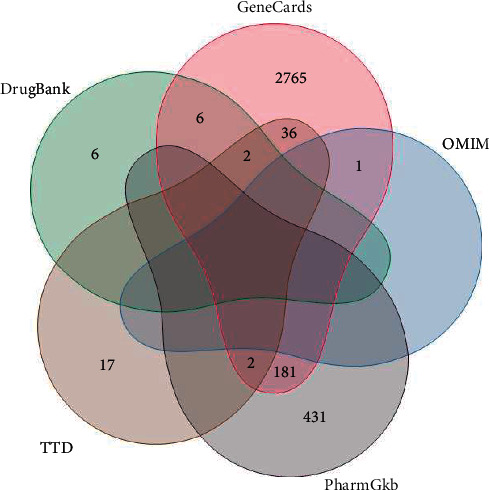
Targets of disease databases.

Based on the GeneCards database, 2993 known depression treatment targets were obtained, while 1 known depression treatment target was obtained based on the OMIM database. There were 614 targets in the PharmGkb database, 57 targets in the TTD database, and 14 targets in the DrugBank database. After eliminating duplicated targets from these five databases, a total of 3447 known depression therapeutic targets were collected. Details can be found in Figure 4. Combined with the GEO dataset and disease database, a total of 3533 depression-related genes were analyzed.

### 3.3. Composite Ingredients and Putative Targets for the Four Herbs

After screening in COREMINE Medical, it was found that the top 4 traditional Chinese medicines with the highest frequency for depression were *Paeonia lactiflora* Pall. (Paeoniaceae, *Paeoniae radix alba*), *Crocus sativus* L. (Iridaceae, croci stigma); *Bupleurum chinense* DC. (Apiaceae, bupleuri radix), and *Cannabis sativa* L. (Cannabaceae, cannabis fructus). *Paeonia lactiflora* Pall. (Paeoniaceae, paeoniae radix alba), sweet in flavor and flat in nature, belongs to the spleen, stomach, and large intestine meridians. *Crocus sativus* L. (Iridaceae, croci stigma), which belongs to the heart and liver meridians, has the effects of promoting blood circulation to remove blood stasis, cooling blood and detoxification, relieving depression, and calming nerves. *Bupleurum chinense* DC. (Apiaceae, bupleuri radix), belonging to the liver, gallbladder, and lung meridians, has the effects of soothing the liver, relieving depression, and raising yang. *Cannabis sativa* L. (Cannabaceae, cannabis fructus), belongs to the liver and spleen meridians, which help to nourish blood and regulate menstruation, suppress yin and stop sweating, soften the liver and relieve pain, and suppress liver yang. Modern research on depression focuses on the stagnation of liver and qi, and emotional disorders; therefore, the four Chinese medicines mentioned above are chosen to perform follow-up network pharmacological analysis.

### 3.4. Known Therapeutic Targets Acting on Depression

A total of 41 chemical ingredients (Supplementary Table S2) of the four herbal medicines were retrieved from TCMSP and related literature: 6 ingredients in *Cannabis sativa* L. (Cannabaceae, cannabis fructus), 5 in *Crocus sativus* L. (Iridaceae, croci stigma), 17 in *Bupleurum chinense* DC. (Apiaceae, bupleuri radix), and 13 in *Paeonia lactiflora* Pall. (Paeoniaceae, paeoniae radix alba). According to the target prediction system in the TCMSP database, we assumed the possible targets of 4 Chinese herbal medicines. As there may be interactions between these medicines and therapies, we eliminated target overlap and obtained 867 target genes of active components; details about the assumed targets are contained in Supplementary Table S3.

### 3.5. Construction of the Drug-Target-Disease Interaction Network

**Figure 5:**
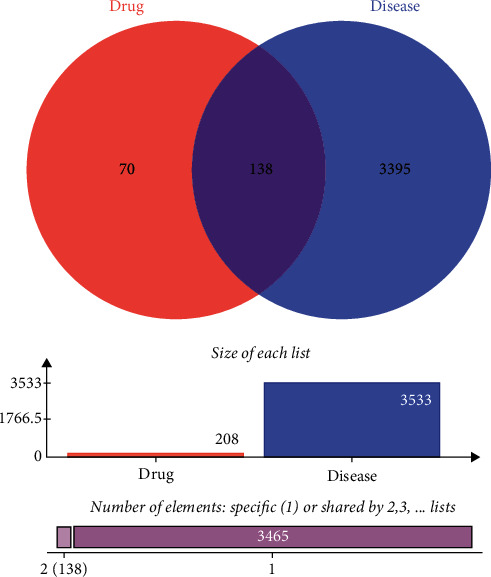
Venn diagram of ingredient-disease target

The construction of a drug-target-disease interaction network led to a total of 138 target genes in the drug-disease intersection, as shown in Figure 5. The intersection of drug targets and depression disease targets in herbs and files of the properties of each node were imported into Cytoscape to construct the network diagram of “drug-target-disease interaction.”

**Figure 6:**
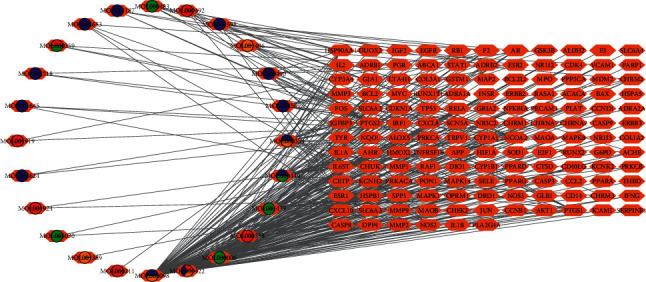
Drug-target-disease network diagram. The green dots represent the hemp kernel's compound molecule. The orange dots represent the molecular compounds in the saffron. The blue dots represent the compound molecules of *Bupleurum*. The red dots represent the compound molecules of *Paeonia paeoniae*. Each edge represents the interaction between the compound molecule and the target.

Different shapes and colors were applied in the network to clearly and intuitively present drug ingredients and the network relationship between drug action targets and disease targets. In the network diagram, different nodes represent drugs, components, and targets, and the wires represent the interaction between the nodes. In this network diagram, there are 24 active ingredients, 122 nodes, and 452 edges of molecules related to drugs, diseases, and compounds, as shown in Figure 6. In the network, the degree value of a node represents the number of routes connected to the nodes in the network. According to the topological properties of the network, the nodes with higher degree values are screened for analysis. These nodes with many connecting compounds or targets act as hubs in the whole network and may be the key compounds or targets.

There were 24 compounds and 138 gene targets in this network, and each compound interacted with an average of 5.75 targets. Each target interacts with an average of 3.3 compounds. Thus, traditional Chinese medicine has various interactions with drugs. Different drugs can have the same molecular compounds. The same compound molecules can be applied to different targets. Different compound molecules can also be applied to the same target. A target can be associated with many diseases. A kind of disease can involve multiple targets and form a complex network.

### 3.6. PPI Network of Therapeutic Targets for Herbs against Depression

**Figure 7:**
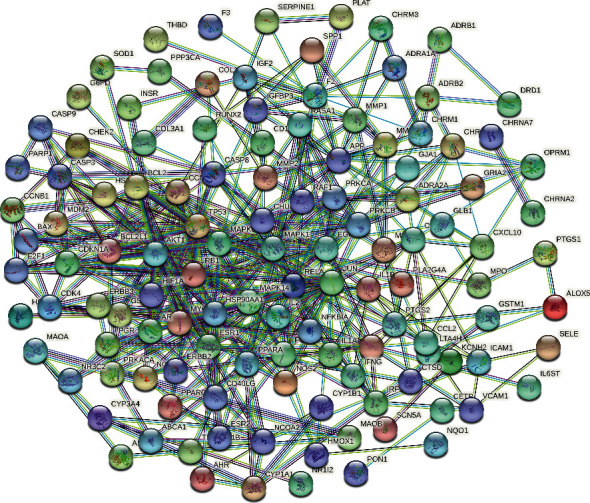
PPI network of constituents, common disease targets.

**Figure 8:**
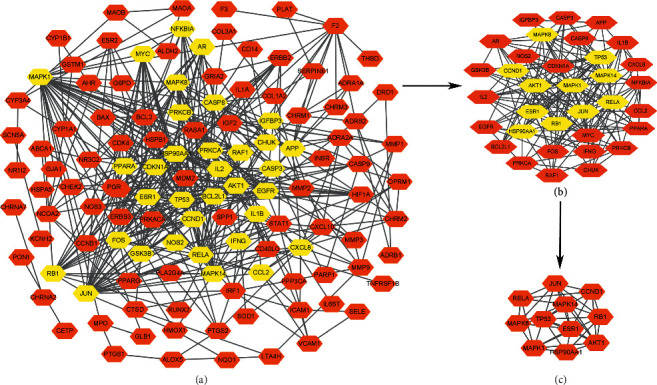
Core targets of HERBS in the treatment of depression.

We screened 141 common drug-disease targets. These targets were put into the Search Tool for the Retrieval of Interacting Genes/Proteins (STRING) database to construct a protein-protein interaction (PPI) network (Figure 7). The minimum interaction threshold is set to “Medium Confidence = 0.9”, and after removing free nodes, the PPI network is obtained. The results obtained from STRING database analysis are shown in Figure 7, where nodes represent proteins, the lines between nodes represent the interactions between two proteins, and different colors correspond to different types of interactions. We applied Cytoscape 3.8.0 and CytoNCA to calculate the mean centrality value of network nodes. We screened the nodes with a centrality value greater than the mean and finally obtained 11 core targets (Figure 8). These core genes included HSP90AA1, AKT1, MAPK1, CCND1, MAPK14, RB1, TP53, JUN, ESR1, RELA, and MAPK8. Results indicated that these targets played an important role in the PPI network and could also be the key targets of herbs in treating depression.

### 3.7. Results of Drug-Disease Intersection Target Genes after GO Analysis

**Figure 9:**
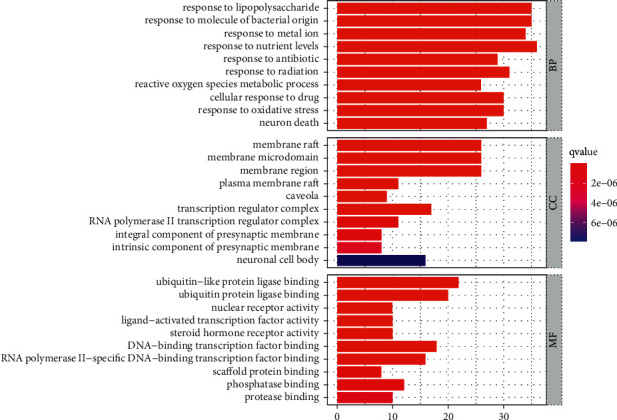
Bar plot of GO analysis results.

To clarify the potential mechanism of herbs acting on depression, the clusterProfiler R package was used to conduct GO analysis at the intersection of drug and disease targets. It included three aspects: biological process (BP), cellular component (CC), and molecular function (MF). A screening standard of *P* < 0.05 was set. Each category was sorted according to the number of enriched genes, and the top 10 items are displayed in the form of a bar graph (Figure 9). A total of 2,207 items were obtained by BP analysis, and the first 10 items were mainly reactions: response to lipopolysaccharide; response to molecule of bacterial origin; response to metal ion; response to nutrient levels; response to antibiotic; response to radiation; reactive oxygen species metabolic process; cellular response to drug; and response to oxidative stress and neuron death. A total of 109 items were obtained by CC analysis, and the first 10 items mainly involved membrane raft; membrane microdomain; membrane region; plasma membrane raft; caveolae; transcription regulator complex; RNA polymerase II transcription regulator complex; integral component of presynaptic membrane; intrinsic component of presynaptic membrane; and neuronal cell body. A total of 197 items were obtained by MF analysis, and the first 10 items were mainly ubiquitin-like protein ligase binding; ubiquitin protein ligase binding; nuclear receptor activity; ligand-activated transcription factor activity; steroid hormone receptor activity; DNA-binding transcription factor binding; RNA polymerase II-specific DNA-binding transcription factor binding; scaffold protein binding; phosphatase binding; and protease binding.

### 3.8. The Results after KEGG Analysis

**Figure 10:**
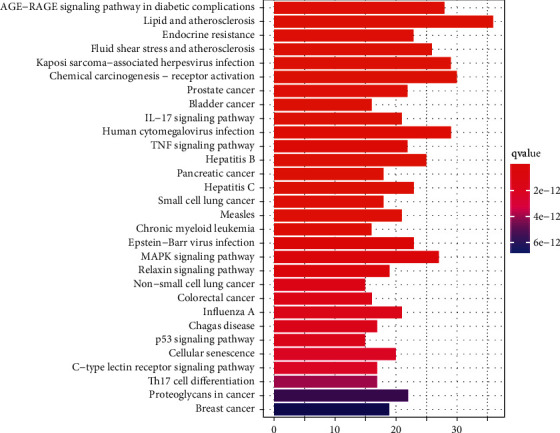
Bar plot of KEGG analysis results.

**Figure 11:**
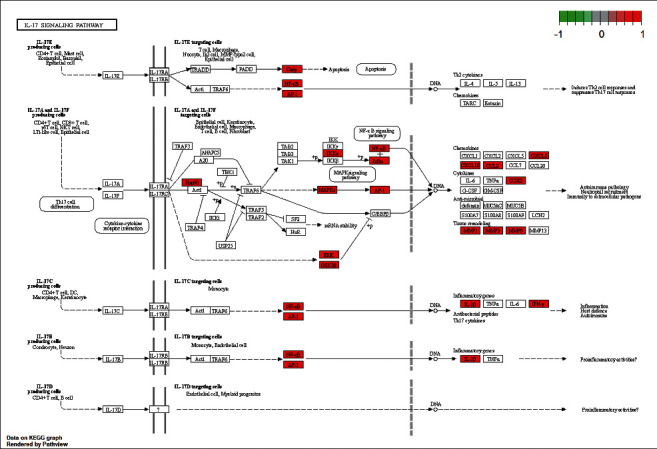
Signaling pathway of IL-17 signaling pathway.

We analyzed the data as well as related biological processes. The clusterProfiler R package was applied to conduct KEGG pathway enrichment analysis on the HERS gene set, and the results are shown (Figures 10 and 11). A total of 180 signaling pathways (*P* < 0.05) were obtained by KEGG pathway enrichment screening, including the IL-17 signaling pathway, apoptosis, pathways of neurodegeneration-multiple diseases, dopaminergic synapse, cell cycle, long-term depression, and neuroactive ligand-receptor interaction.  Figure 10 shows the top 30 paths sorted from smallest to largest by *P* value.  Figure 11 shows the signaling pathway of long-term depression.

### 3.9. Molecular Docking

**Figure 12:**
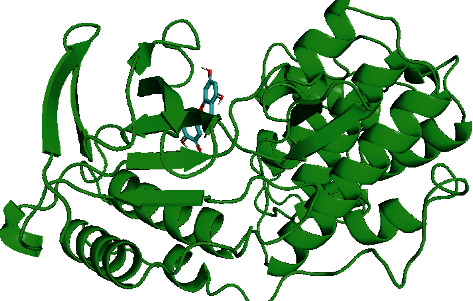
Docked conformation of MAPK14 and quercetin.

To ensure that the accuracy of this study is within the acceptable level, we applied iGEM-DOCK molecular docking analysis to conduct molecular docking between 9 core genes of herbs acting on depression and the key effective compounds obtained by topological analysis. The more stable the binding structure of the small molecule receptor and ligand, the lower the energy level, and the larger the interaction type should be. According to the docking score (Table 2), MAPK14 and petunidin, MAPK14 and isorhamnetin, and MAPK1 and quercetin had the strongest combination and a low energy score. The specific docking structure is shown in Figure 12.

## 4. Discussion

Depression belongs to the category of “melancholia” and “zang manic” in Chinese medicine. Through the analysis of COREMINE Medical, we can know that hemp seed, saffron, *Bupleurum*, *Cannabis sativa* L. (Cannabaceae, cannabis fructus), and *Crocus sativus* L. (Iridaceae, croci stigma), four traditional Chinese medicines, can target the key genes of depression. It has a certain antidepressive effect and multitarget and multilevel effects. Hemp oil can partially improve the depressive behavior and cognitive ability of mice and reduce the expression of IL-1*β*. The inflammatory response of hemp seed oil has a certain effect on depression mice [19]. *Bupleurum chinense* DC. (Apiaceae, bupleuri radix) and radix paeoniae alba in antidepressant research are more, to explore the medication rules of traditional Chinese medicine in treating poststroke depression by “traditional Chinese medicine inheritance support system (V2.5).” The results showed that *Bupleurum chinense* DC. (Apiaceae, bupleuri radix) and Radix Paeoniae Alba were the top five Chinese medicines [26]. Vahdati Hassani et al. [27] suggested that crocin has antidepressant-like action by increasing CREB, BDNF, and VGF levels in the hippocampus. Zhang et al. [28] showed that crocin attenuated LPS-induced production of reactive oxygen species and microglial M1-polarization. Moreover, crocin inhibited LPS-induced anxiety and depressive-like behaviors in mice. Crocin blocked the effect of increased cytokine expression including IL-1*β*, IL-18, and TNF-*α* in the hippocampus of LPS-injected mice. Additionally, LPS-injected mice exhibited elevated expression of NF-*κ*B, p65, NLRP3, caspase-1, and the adaptor protein ASC, which were inhibited by crocin treatment. In summary, crocin attenuates LPS-induced anxiety and depressive-like behaviors by suppressing the NF-kB and NLRP3 signaling pathways.

In this study, network pharmacological methods were used to explore the possible targets and pathways of *Cannabis sativa* L. (Cannabaceae, cannabis fructus), *Crocus sativus* L. (Iridaceae, croci stigma), *Bupleurum chinense* DC. (Apiaceae, bupleuri radix), and *Paeonia lactiflora* Pall. (Paeoniaceae, paeoniae radix alba). Among the selected 24 compounds corresponding to 138 action targets, the top 5 compounds with the strongest effect were finally analyzed, including petunidin, isorhamnetin, luteolin, quercetin, and kaempferol. At present, there are few studies on morning-glory as an antidepressant, and morning-glory is likely to be a targeted drug for the treatment of depression. Petunidin belongs to *Bupleurum chinense* DC. (Apiaceae, bupleuri radix). Isorhamnetin belongs to the *Bupleurum chinense* DC. (Apiaceae, bupleuri radix) and *Crocus sativus* L. (Iridaceae, croci stigma). Luteolin belongs to *Cannabis sativa* L. (Cannabaceae, cannabis fructus). Quercetin belongs to *Bupleurum chinense* DC. (Apiaceae, bupleuri radix), and *Crocus sativus* L. (Iridaceae, croci stigma). Kaempferol belongs to *Bupleurum chinense* DC. (Apiaceae, bupleuri radix), *Crocus sativus* L. (Iridaceae, croci stigma), and *Paeonia lactiflora* Pall. (Paeoniaceae, paeoniae radix alba). It has been reported that isorhamnetin, kaempferol, and quercetin have antidepressant effects [29, 30]. Luteolin can improve depression-like behavior in CUMS mice, the mechanism of which may be related to the enhancement of antioxidant activity and the improvement of oxidative/antioxidant balance in brain tissue of mice [31]. Quercetin can significantly reverse corticosterone-releasing factor- (CRF-) induced anxiety and depression-like behavior in rats [32]. Kaempferol can enhance the antioxidant capacity of the cerebral cortex and upregulate the activity of the Akt/*β*-catenin cascade to induce anti-inflammatory effect and then play an antidepressant role in chronic social defeat stress (CSDS) mice [33]. This also provides a theoretical basis for the rationality of these four herbs in the treatment of depression.

According to the target of ingredient-disease intersection, these four traditional Chinese medicines can act on multiple targets of HSP90AA1, Akt1, MAPK1, CCND1, MAPK14, RB1, TP53, Jun, ESR1, Rela, and MAPK8 to produce synergistic antidepressant effects. GO and KEGG analysis revealed that the mechanism of depression treatment may be through response to oxidative stress and neuron death, steroid hormone receptor activity; IL-17 signaling pathway, apoptosis, pathways of neurodegeneration-multiple diseases, dopaminergic synapse, cell cycle, long-term depression, neuroactive ligand-receptor interaction play a role in improving the effect of depression. MAPKs are the core targets involved in the IL-17 signaling pathway. The possible effects are petunidin on MAPK14, isorhamnetin on MAPK14, quercetin on MAPK1, and luteolin on MAPK1.

Astrocytic p38*α* MAPK drives NMDA receptor-dependent long-term depression and modulates long-term memory [34]. Serotonin facilitates long-term depression induction in the prefrontal cortex via p38 MAPK/Rab5-mediated enhancement of AMPA receptor internalization [35]. MAPK signaling determines anxiety in the Juvenile Mouse brain but depression-like behavior in adults [36]. Enhanced MAPK1 function causes a neurodevelopmental disorder within the RASopathy clinical spectrum [37]. The P2RX7-MAPK1/2-SP1 axis inhibits MTOR independent HSPB1-mediated astroglial autophagy [38]. Enhanced MAPK1 function causes a neurodevelopmental disorder within the RASopathy clinical spectrum [37]. The P2RX7-MAPK1/2-SP1 axis inhibits MTOR independent HSPB1-mediated astroglial autophagy [38]. The mechanism of Alzheimer's disease is that MAPK14/P38A activates the targeting neurons to regulate autophagy. Mitophagy is primarily due to alternative autophagy and requires the MAPK1 and MAPK14 signaling pathways [39, 40]. Depression is accompanied by increased IL-17 mRNA and protein levels in serum, thereby confirming the important role of IL-17 production in depression and its possible use as a biomarker of depression [41]. However, adipose tissue also expresses high levels of circulating interleukin-17 (IL-17A), mainly secreted by T helper 17 (Th17, CD4+) cells [42–44]. In addition to their local action, systemic cytokines derived from adipose tissue may also enter the central nervous system which leads to neuroinflammation [45]. Inflammatory factors cause excessive activation of IDO (indoleamine-2,3-dioxygenase), an enzyme present in microglia, astrocytes and neurons, and catabolizing the conversion of tryptophan into the neurotoxic kynurenine (KYN), thus reducing the availability of tryptophan for the production of serotonin. Kynurenine, in turn, influences the intensification of neurodegenerative processes [46, 47]. Altered neurogenesis and neuroplasticity and serotonin deficits contribute to depression. MAPK14, as an Alzheimer's therapeutic target, has otherwise primarily been considered as an anti-inflammatory mechanism to target innate immune responses in the brain [48, 49], particularly with regard to microglial activation, as well as reducing inflammation-induced synaptic toxicity. Quercetin alleviates anxiety and depression in 3-nitropropionic acid-induced Huntington's disease in rats [50]. Quercetin alleviates LPS-induced depression-like behavior in rats by regulating BDNF-related imbalance of copine 6 and TREM1/2 in the hippocampus and PFC [51].

## 5. Conclusion

Overall, this study systematically summarized key active compounds in *Paeonia lactiflora* Pall. (Paeoniaceae, paeoniae radix alba), *Crocus sativus* L. (Iridaceae, croci stigma), *Bupleurum chinense* DC. (Apiaceae, bupleuri radix), and *Cannabis sativa* L. (Cannabaceae, cannabis fructus) and comprehensively analyzed potential mechanisms of its action and pathways. The current findings revealed the complicated antidepressant mechanism of most commonly used Chinese medicines and provided a rational strategy for revealing the complex composition and function of Chinese herbal formulas.

## Figures and Tables

**Table 1:** Information of GEO datasets.

Number	The dataset	Link of the GEO database
1	GSE53987-HPC	https://www.ncbi.nlm.nih.gov/geo/query/acc.cgi?acc=GSE53987
2	GSE53987-STR	https://www.ncbi.nlm.nih.gov/geo/query/acc.cgi?acc=GSE53987
3	GSE53987-PFC	https://www.ncbi.nlm.nih.gov/geo/query/acc.cgi?acc=GSE53987
4	GSE54562	https://www.ncbi.nlm.nih.gov/geo/query/acc.cgi?acc=GSE54562
5	GSE54563	https://www.ncbi.nlm.nih.gov/geo/query/acc.cgi?acc=GSE54563
6	GSE54564	https://www.ncbi.nlm.nih.gov/geo/query/acc.cgi?acc=GSE54564
7	GSE54565	https://www.ncbi.nlm.nih.gov/geo/query/acc.cgi?acc=GSE54565
8	GSE54566	https://www.ncbi.nlm.nih.gov/geo/query/acc.cgi?acc=GSE54566
9	GSE54567	https://www.ncbi.nlm.nih.gov/geo/query/acc.cgi?acc=GSE54567
10	GSE54568	https://www.ncbi.nlm.nih.gov/geo/query/acc.cgi?acc=GSE54568
11	GSE54570	https://www.ncbi.nlm.nih.gov/geo/query/acc.cgi?acc=GSE54570
12	GSE54571	https://www.ncbi.nlm.nih.gov/geo/query/acc.cgi?acc=GSE54571
13	GSE54572	https://www.ncbi.nlm.nih.gov/geo/query/acc.cgi?acc=GSE54572
14	GSE54575	https://www.ncbi.nlm.nih.gov/geo/query/acc.cgi?acc=GSE54575
15	GSE12654	https://www.ncbi.nlm.nih.gov/geo/query/acc.cgi?acc=GSE12654

**Table 2:** Molecular docking score.

Target	PDB ID	Compound	Affinity (kJ·mol^−1^)
JUN	P05412	Luteolin	−8.8
		Quercetin	−8.6
		Kaempferol	−8.4
		Beta-sitosterol	−6.8
MAPK8	P45983	Kaempferol	−5.5
RELA	P9WHG9	Isorhamnetin	−7.6
		Quercetin	−7.5
		Arachidonic acid	−5
TP53	Q96S44	Luteolin	−8.2
		Quercetin	−8
RB1	P13405	Quercetin	−8.4
		Luteolin	−8.9
MAPK14	Q16539	Petunidin	−9
		Isorhamnetin	−9
CCND1	*P24385*	Quercetin	−7.1
		Arachidonic acid	−4.9
		Luteolin	−7.3
MAPK1	P28482	Quercetin	-8.4
		Arachidonic acid	−5.8
		Luteolin	−8.5
AKT1	P31750	Luteolin	−6.3
		Quercetin	−6.3
		Kaempferol	−6.4
HSP90AA1	P07900	Kaempferol	−6.7
		Beta-sitosterol	−7.4
		(+)-Catechin	−6.8
		Isorhamnetin	−6.9
		Cubebin	−6.7
		Quercetin	−6.9
ESR1	P03372	(+)-Catechin	−7.1
		Isorhamnetin	−8.5

## Data Availability

The data used to support the findings of this study are available from the corresponding author upon request.

## Supplementary Materials

Supplementary Materials Table S1: database and website. Table S2: ingredients. Table S3: targets.
