# Supplementary material for: Identification of Potential Biomarkers of Depression and Network Pharmacology Approach to Investigate the Mechanism of Key Genes and Therapeutic Traditional Chinese Medicine in the Treatment of Depression
Source: Evid Based Complement Alternat Med. 2021 Dec 31;2021:2165632. doi: 10.1155/2021/2165632 (PMC8741373; doi:10.1155/2021/2165632)
Supplement: Supplementary Materials — Table S1: database and website. Table S2: ingredients. Table S3: targets. [file 2165632.f1.docx]

| Table S1 Database and website | |
| --- | --- |
| Database | Website |
| DrugBank | <https://go.drugbank.com/> |
| GeneCards | <https://www.genecards.org/> |
| OMIM | <https://www.omim.org/> |
| PharmGkb | <https://www.pharmgkb.org/> |
| TTD | <http://db.idrblab.net/ttd/> |
| TCMSP | <https://tcmsp-e.com/> |
| STRING | https://string-db.org/ |

| Table S2 Ingredients | | | | |
| --- | --- | --- | --- | --- |
| Herbs | Mol ID | Molecule Name | OB(%) | DL |
| Cannabis sativa L. [Cannabaceae, cannabis fructus] | MOL001439 | arachidonic acid | 45.57324991 | 0.20409 |
|  | MOL000359 | sitosterol | 36.91390583 | 0.7512 |
|  | MOL000449 | Stigmasterol | 43.82985158 | 0.75665 |
|  | MOL000483 | (Z)-3-(4-hydroxy-3-methoxy-phenyl)-N-[2-(4-hydroxyphenyl)ethyl]acrylamide | 118.3477485 | 0.26399 |
|  | MOL005030 | gondoic acid | 30.70294255 | 0.19743 |
|  | MOL000006 | luteolin | 36.16262934 | 0.24552 |
| Crocus sativus L. [Iridaceae, croci stigma] | MOL001389 | n-heptanal | 79.73518386 | 0.59283 |
|  | MOL001406 | crocetin | 35.29636648 | 0.25639 |
|  | MOL000354 | isorhamnetin | 49.60437705 | 0.306 |
|  | MOL000422 | kaempferol | 41.88224954 | 0.24066 |
|  | MOL000098 | quercetin | 46.43334812 | 0.27525 |
| Bupleurum chinense DC. [Apiaceae, bupleuri radix] | MOL000490 | petunidin | 30.04553904 | 0.30712 |
|  | MOL004702 | saikosaponin c_qt | 30.5049277 | 0.63193 |
|  | MOL004648 | Troxerutin | 31.59657437 | 0.28256 |
|  | MOL004598 | 3,5,6,7-tetramethoxy-2-(3,4,5-trimethoxyphenyl)chromone | 31.97495927 | 0.59317 |
|  | MOL002776 | Baicalin | 40.12360996 | 0.75264 |
|  | MOL000422 | kaempferol | 41.88224954 | 0.24066 |
|  | MOL001645 | Linoleyl acetate | 42.10076623 | 0.19845 |
|  | MOL004718 | α-spinasterol | 42.97936552 | 0.75693 |
|  | MOL000449 | Stigmasterol | 43.82985158 | 0.75665 |
|  | MOL004653 | (+)-Anomalin | 46.05534076 | 0.6566 |
|  | MOL000098 | quercetin | 46.43334812 | 0.27525 |
|  | MOL004624 | Longikaurin A | 47.72214984 | 0.53015 |
|  | MOL004628 | Octalupine | 47.8222494 | 0.27864 |
|  | MOL004609 | Areapillin | 48.96435072 | 0.41394 |
|  | MOL000354 | isorhamnetin | 49.60437705 | 0.306 |
|  | MOL013187 | Cubebin | 57.1281289 | 0.63988 |
|  | MOL004644 | Sainfuran | 79.90979196 | 0.23331 |
| Paeonia lactiflora Pall. [Paeoniaceae, paeoniae radix alba] | MOL001930 | benzoyl paeoniflorin | 31.27447298 | 0.74612 |
|  | MOL000359 | sitosterol | 36.91390583 | 0.7512 |
|  | MOL000358 | beta-sitosterol | 36.91390583 | 0.75123 |
|  | MOL000422 | kaempferol | 41.88224954 | 0.24066 |
|  | MOL001919 | (3S,5R,8R,9R,10S,14S)-3,17-dihydroxy-4,4,8,10,14-pentamethyl-2,3,5,6,7,9-hexahydro-1H-cyclopenta[a]phenanthrene-15,16-dione | 43.55620167 | 0.53276 |
|  | MOL001921 | Lactiflorin | 49.12131675 | 0.79711 |
|  | MOL001924 | paeoniflorin | 53.87037516 | 0.78709 |
|  | MOL000492 | (+)-catechin | 54.82643405 | 0.24164 |
|  | MOL000211 | Mairin | 55.37707338 | 0.7761 |
|  | MOL001910 | 11alpha,12alpha-epoxy-3beta-23-dihydroxy-30-norolean-20-en-28,12beta-olide | 64.77389307 | 0.37586 |
|  | MOL001928 | albiflorin_qt | 66.64076901 | 0.32626 |
|  | MOL001925 | paeoniflorin_qt | 68.17576188 | 0.39507 |
|  | MOL001918 | paeoniflorgenone | 87.59312084 | 0.36678 |

| Table S3 Targets | | | |
| --- | --- | --- | --- |
| Drug | MolId | MolName | Target |
| Paeonia lactiflora Pall. [Paeoniaceae, paeoniae radix alba] | Mol ID | Molecule Name | Target Name |
|  | MOL001918 | paeoniflorgenone | Gamma-aminobutyric-acid receptor subunit alpha-1 |
|  | MOL001919 | (3S,5R,8R,9R,10S,14S)-3,17-dihydroxy-4,4,8,10,14-pentamethyl-2,3,5,6,7,9-hexahydro-1H-cyclopenta[a]phenanthrene-15,16-dione | Progesterone receptor |
|  | MOL001919 | (3S,5R,8R,9R,10S,14S)-3,17-dihydroxy-4,4,8,10,14-pentamethyl-2,3,5,6,7,9-hexahydro-1H-cyclopenta[a]phenanthrene-15,16-dione | Mineralocorticoid receptor |
|  | MOL001924 | paeoniflorin | Tumor necrosis factor |
|  | MOL001924 | paeoniflorin | Interleukin-6 |
|  | MOL001924 | paeoniflorin | Monocyte differentiation antigen CD14 |
|  | MOL001924 | paeoniflorin | Lipopolysaccharide-binding protein |
|  | MOL000211 | Mairin | Progesterone receptor |
|  | MOL000358 | beta-sitosterol | Progesterone receptor |
|  | MOL000358 | beta-sitosterol | Nuclear receptor coactivator 2 |
|  | MOL000358 | beta-sitosterol | Prostaglandin G/H synthase 1 |
|  | MOL000358 | beta-sitosterol | Prostaglandin G/H synthase 2 |
|  | MOL000358 | beta-sitosterol | Heat shock protein HSP 90-alpha |
|  | MOL000358 | beta-sitosterol | Phosphatidylinositol-4,5-bisphosphate 3-kinase catalytic subunit gamma isoform |
|  | MOL000358 | beta-sitosterol | Potassium voltage-gated channel subfamily H member 2 |
|  | MOL000358 | beta-sitosterol | cAMP-dependent protein kinase catalytic subunit alpha |
|  | MOL000358 | beta-sitosterol | D(1A) dopamine receptor |
|  | MOL000358 | beta-sitosterol | Muscarinic acetylcholine receptor M3 |
|  | MOL000358 | beta-sitosterol | Muscarinic acetylcholine receptor M1 |
|  | MOL000358 | beta-sitosterol | Sodium channel protein type 5 subunit alpha |
|  | MOL000358 | beta-sitosterol | Gamma-aminobutyric-acid receptor subunit alpha-2 |
|  | MOL000358 | beta-sitosterol | Muscarinic acetylcholine receptor M4 |
|  | MOL000358 | beta-sitosterol | cGMP-inhibited 3',5'-cyclic phosphodiesterase A |
|  | MOL000358 | beta-sitosterol | 5-hydroxytryptamine 2A receptor |
|  | MOL000358 | beta-sitosterol | Gamma-aminobutyric-acid receptor subunit alpha-5 |
|  | MOL000358 | beta-sitosterol | Alpha-1A adrenergic receptor |
|  | MOL000358 | beta-sitosterol | Gamma-aminobutyric-acid receptor subunit alpha-3 |
|  | MOL000358 | beta-sitosterol | Muscarinic acetylcholine receptor M2 |
|  | MOL000358 | beta-sitosterol | Alpha-1B adrenergic receptor |
|  | MOL000358 | beta-sitosterol | Beta-2 adrenergic receptor |
|  | MOL000358 | beta-sitosterol | Neuronal acetylcholine receptor subunit alpha-2 |
|  | MOL000358 | beta-sitosterol | Sodium-dependent serotonin transporter |
|  | MOL000358 | beta-sitosterol | Mu-type opioid receptor |
|  | MOL000358 | beta-sitosterol | Gamma-aminobutyric-acid receptor subunit alpha-1 |
|  | MOL000358 | beta-sitosterol | Neuronal acetylcholine receptor subunit alpha-7 |
|  | MOL000358 | beta-sitosterol | Cytochrome P450-cam |
|  | MOL000358 | beta-sitosterol | Apoptosis regulator Bcl-2 |
|  | MOL000358 | beta-sitosterol | Apoptosis regulator BAX |
|  | MOL000358 | beta-sitosterol | Caspase-9 |
|  | MOL000358 | beta-sitosterol | Transcription factor AP-1 |
|  | MOL000358 | beta-sitosterol | Caspase-3 |
|  | MOL000358 | beta-sitosterol | Caspase-8 |
|  | MOL000358 | beta-sitosterol | Protein kinase C alpha type |
|  | MOL000358 | beta-sitosterol | Transforming growth factor beta-1 |
|  | MOL000358 | beta-sitosterol | Serum paraoxonase/arylesterase 1 |
|  | MOL000358 | beta-sitosterol | Microtubule-associated protein 2 |
|  | MOL000359 | sitosterol | Progesterone receptor |
|  | MOL000359 | sitosterol | Nuclear receptor coactivator 2 |
|  | MOL000359 | sitosterol | Mineralocorticoid receptor |
|  | MOL000422 | kaempferol | Nitric oxide synthase, inducible |
|  | MOL000422 | kaempferol | Prostaglandin G/H synthase 1 |
|  | MOL000422 | kaempferol | Androgen receptor |
|  | MOL000422 | kaempferol | Peroxisome proliferator-activated receptor gamma |
|  | MOL000422 | kaempferol | Prostaglandin G/H synthase 2 |
|  | MOL000422 | kaempferol | Heat shock protein HSP 90-alpha |
|  | MOL000422 | kaempferol | Phosphatidylinositol-4,5-bisphosphate 3-kinase catalytic subunit gamma isoform |
|  | MOL000422 | kaempferol | cAMP-dependent protein kinase catalytic subunit alpha |
|  | MOL000422 | kaempferol | Nuclear receptor coactivator 2 |
|  | MOL000422 | kaempferol | Dipeptidyl peptidase 4 |
|  | MOL000422 | kaempferol | Trypsin-1 |
|  | MOL000422 | kaempferol | Progesterone receptor |
|  | MOL000422 | kaempferol | Prothrombin |
|  | MOL000422 | kaempferol | Muscarinic acetylcholine receptor M1 |
|  | MOL000422 | kaempferol | Nitric-oxide synthase, endothelial |
|  | MOL000422 | kaempferol | Gamma-aminobutyric-acid receptor subunit alpha-2 |
|  | MOL000422 | kaempferol | Acetylcholinesterase |
|  | MOL000422 | kaempferol | Sodium-dependent noradrenaline transporter |
|  | MOL000422 | kaempferol | Muscarinic acetylcholine receptor M2 |
|  | MOL000422 | kaempferol | Alpha-1B adrenergic receptor |
|  | MOL000422 | kaempferol | Gamma-aminobutyric-acid receptor subunit alpha-1 |
|  | MOL000422 | kaempferol | DNA topoisomerase 2-alpha |
|  | MOL000422 | kaempferol | Coagulation factor VII |
|  | MOL000422 | kaempferol | Calmodulin |
|  | MOL000422 | kaempferol | Transcription factor p65 |
|  | MOL000422 | kaempferol | Inhibitor of nuclear factor kappa-B kinase subunit beta |
|  | MOL000422 | kaempferol | RAC-alpha serine/threonine-protein kinase |
|  | MOL000422 | kaempferol | Apoptosis regulator Bcl-2 |
|  | MOL000422 | kaempferol | Apoptosis regulator BAX |
|  | MOL000422 | kaempferol | Tumor necrosis factor |
|  | MOL000422 | kaempferol | Transcription factor AP-1 |
|  | MOL000422 | kaempferol | Activator of 90 kDa heat shock protein ATPase homolog 1 |
|  | MOL000422 | kaempferol | Caspase-3 |
|  | MOL000422 | kaempferol | Mitogen-activated protein kinase 8 |
|  | MOL000422 | kaempferol | Xanthine dehydrogenase/oxidase |
|  | MOL000422 | kaempferol | Interstitial collagenase |
|  | MOL000422 | kaempferol | Signal transducer and activator of transcription 1-alpha/beta |
|  | MOL000422 | kaempferol | Cell division control protein 2 homolog |
|  | MOL000422 | kaempferol | Peroxisome proliferator-activated receptor gamma |
|  | MOL000422 | kaempferol | Heme oxygenase 1 |
|  | MOL000422 | kaempferol | Cytochrome P450 3A4 |
|  | MOL000422 | kaempferol |  |
|  | MOL000422 | kaempferol | Cytochrome P450 1A1 |
|  | MOL000422 | kaempferol | Intercellular adhesion molecule 1 |
|  | MOL000422 | kaempferol | E-selectin |
|  | MOL000422 | kaempferol | Vascular cell adhesion protein 1 |
|  | MOL000422 | kaempferol | Nuclear receptor subfamily 1 group I member 2 |
|  | MOL000422 | kaempferol | Cytochrome P450 1B1 |
|  | MOL000422 | kaempferol | Arachidonate 5-lipoxygenase |
|  | MOL000422 | kaempferol | Hyaluronan synthase 2 |
|  | MOL000422 | kaempferol | Aryl hydrocarbon receptor |
|  | MOL000422 | kaempferol | 26S proteasome non-ATPase regulatory subunit 3 |
|  | MOL000422 | kaempferol | Solute carrier family 2, facilitated glucose transporter member 4 |
|  | MOL000422 | kaempferol | Nuclear receptor subfamily 1 group I member 3 |
|  | MOL000422 | kaempferol | Insulin receptor |
|  | MOL000422 | kaempferol | Type I iodothyronine deiodinase |
|  | MOL000422 | kaempferol | Serine/threonine-protein phosphatase 2B catalytic subunit alpha isoform |
|  | MOL000422 | kaempferol | Peroxidase C1A |
|  | MOL000422 | kaempferol | Glutathione S-transferase Mu 1 |
|  | MOL000422 | kaempferol | Glutathione S-transferase Mu 2 |
|  | MOL000422 | kaempferol | Aldo-keto reductase family 1 member C3 |
|  | MOL000422 | kaempferol | Antileukoproteinase |
|  | MOL000492 | (+)-catechin | Prostaglandin G/H synthase 1 |
|  | MOL000492 | (+)-catechin | Estrogen receptor |
|  | MOL000492 | (+)-catechin | Prostaglandin G/H synthase 2 |
|  | MOL000492 | (+)-catechin | Heat shock protein HSP 90-alpha |
|  | MOL000492 | (+)-catechin | Beta-lactamase |
|  | MOL000492 | (+)-catechin | cAMP-dependent protein kinase catalytic subunit alpha |
|  | MOL000492 | (+)-catechin | Nuclear receptor coactivator 2 |
|  | MOL000492 | (+)-catechin | Calmodulin |
|  | MOL000492 | (+)-catechin | Retinoic acid receptor RXR-alpha |
|  | MOL000492 | (+)-catechin | Hyaluronan synthase 2 |
| Bupleurum chinense DC. [Apiaceae, bupleuri radix] | Mol ID | Molecule Name | Target Name |
|  | MOL001645 | Linoleyl acetate | Prostaglandin G/H synthase 1 |
|  | MOL001645 | Linoleyl acetate | Prostaglandin G/H synthase 2 |
|  | MOL001645 | Linoleyl acetate | Nuclear receptor coactivator 2 |
|  | MOL001645 | Linoleyl acetate | Retinoic acid receptor RXR-alpha |
|  | MOL002776 | Baicalin | Coagulation factor X |
|  | MOL002776 | Baicalin | Tyrosine-protein phosphatase non-receptor type 1 |
|  | MOL000449 | Stigmasterol | Progesterone receptor |
|  | MOL000449 | Stigmasterol | Mineralocorticoid receptor |
|  | MOL000449 | Stigmasterol | Nuclear receptor coactivator 2 |
|  | MOL000449 | Stigmasterol |  |
|  | MOL000449 | Stigmasterol | Ig gamma-1 chain C region |
|  | MOL000449 | Stigmasterol | Retinoic acid receptor RXR-alpha |
|  | MOL000449 | Stigmasterol | Nuclear receptor coactivator 1 |
|  | MOL000449 | Stigmasterol | Prostaglandin G/H synthase 1 |
|  | MOL000449 | Stigmasterol | Prostaglandin G/H synthase 2 |
|  | MOL000449 | Stigmasterol | Alpha-2A adrenergic receptor |
|  | MOL000449 | Stigmasterol | Sodium-dependent noradrenaline transporter |
|  | MOL000449 | Stigmasterol | Sodium-dependent dopamine transporter |
|  | MOL000449 | Stigmasterol | Beta-2 adrenergic receptor |
|  | MOL000449 | Stigmasterol | Aldose reductase |
|  | MOL000449 | Stigmasterol | Urokinase-type plasminogen activator |
|  | MOL000449 | Stigmasterol | Leukotriene A-4 hydrolase |
|  | MOL000449 | Stigmasterol | Amine oxidase [flavin-containing] B |
|  | MOL000449 | Stigmasterol | Amine oxidase [flavin-containing] A |
|  | MOL000449 | Stigmasterol | cAMP-dependent protein kinase catalytic subunit alpha |
|  | MOL000449 | Stigmasterol | Chymotrypsinogen B |
|  | MOL000449 | Stigmasterol | Muscarinic acetylcholine receptor M3 |
|  | MOL000449 | Stigmasterol | Muscarinic acetylcholine receptor M1 |
|  | MOL000449 | Stigmasterol | Beta-1 adrenergic receptor |
|  | MOL000449 | Stigmasterol | Sodium channel protein type 5 subunit alpha |
|  | MOL000449 | Stigmasterol | 5-hydroxytryptamine 2A receptor |
|  | MOL000449 | Stigmasterol | Alpha-1A adrenergic receptor |
|  | MOL000449 | Stigmasterol | Gamma-aminobutyric-acid receptor subunit alpha-3 |
|  | MOL000449 | Stigmasterol | Muscarinic acetylcholine receptor M2 |
|  | MOL000449 | Stigmasterol | Alpha-1B adrenergic receptor |
|  | MOL000449 | Stigmasterol | Gamma-aminobutyric-acid receptor subunit alpha-1 |
|  | MOL000449 | Stigmasterol | Neuronal acetylcholine receptor subunit alpha-7 |
|  | MOL000354 | isorhamnetin | Nitric oxide synthase, inducible |
|  | MOL000354 | isorhamnetin | Prostaglandin G/H synthase 1 |
|  | MOL000354 | isorhamnetin | Estrogen receptor |
|  | MOL000354 | isorhamnetin | Androgen receptor |
|  | MOL000354 | isorhamnetin | Peroxisome proliferator-activated receptor gamma |
|  | MOL000354 | isorhamnetin | Prostaglandin G/H synthase 2 |
|  | MOL000354 | isorhamnetin | Tyrosine-protein phosphatase non-receptor type 1 |
|  | MOL000354 | isorhamnetin | Estrogen receptor beta |
|  | MOL000354 | isorhamnetin | Dipeptidyl peptidase 4 |
|  | MOL000354 | isorhamnetin | Mitogen-activated protein kinase 14 |
|  | MOL000354 | isorhamnetin | Glycogen synthase kinase-3 beta |
|  | MOL000354 | isorhamnetin | Heat shock protein HSP 90-alpha |
|  | MOL000354 | isorhamnetin | Cell division protein kinase 2 |
|  | MOL000354 | isorhamnetin | Phosphatidylinositol-4,5-bisphosphate 3-kinase catalytic subunit gamma isoform |
|  | MOL000354 | isorhamnetin | cAMP-dependent protein kinase catalytic subunit alpha |
|  | MOL000354 | isorhamnetin | Trypsin-1 |
|  | MOL000354 | isorhamnetin | Proto-oncogene serine/threonine-protein kinase Pim-1 |
|  | MOL000354 | isorhamnetin | Cyclin-A2 |
|  | MOL000354 | isorhamnetin | Nuclear receptor coactivator 2 |
|  | MOL000354 | isorhamnetin | Calmodulin |
|  | MOL000354 | isorhamnetin | Glycogen phosphorylase, muscle form |
|  | MOL000354 | isorhamnetin | Peroxisome proliferator-activated receptor delta |
|  | MOL000354 | isorhamnetin | Serine/threonine-protein kinase Chk1 |
|  | MOL000354 | isorhamnetin | Aldose reductase |
|  | MOL000354 | isorhamnetin | Nuclear receptor coactivator 1 |
|  | MOL000354 | isorhamnetin | Coagulation factor VII |
|  | MOL000354 | isorhamnetin | Prothrombin |
|  | MOL000354 | isorhamnetin | Nitric-oxide synthase, endothelial |
|  | MOL000354 | isorhamnetin | Acetylcholinesterase |
|  | MOL000354 | isorhamnetin | Gamma-aminobutyric-acid receptor subunit alpha-1 |
|  | MOL000354 | isorhamnetin | Amine oxidase [flavin-containing] B |
|  | MOL000354 | isorhamnetin | Glutamate receptor 2 |
|  | MOL000354 | isorhamnetin | Cytochrome P450-cam |
|  | MOL000354 | isorhamnetin | Transcription factor p65 |
|  | MOL000354 | isorhamnetin | Xanthine dehydrogenase/oxidase |
|  | MOL000354 | isorhamnetin | Neutrophil cytosol factor 1 |
|  | MOL000354 | isorhamnetin | Oxidized low-density lipoprotein receptor 1 |
|  | MOL000422 | kaempferol | Nitric oxide synthase, inducible |
|  | MOL000422 | kaempferol | Prostaglandin G/H synthase 1 |
|  | MOL000422 | kaempferol | Androgen receptor |
|  | MOL000422 | kaempferol | Peroxisome proliferator-activated receptor gamma |
|  | MOL000422 | kaempferol | Prostaglandin G/H synthase 2 |
|  | MOL000422 | kaempferol | Heat shock protein HSP 90-alpha |
|  | MOL000422 | kaempferol | Phosphatidylinositol-4,5-bisphosphate 3-kinase catalytic subunit gamma isoform |
|  | MOL000422 | kaempferol | cAMP-dependent protein kinase catalytic subunit alpha |
|  | MOL000422 | kaempferol | Nuclear receptor coactivator 2 |
|  | MOL000422 | kaempferol | Dipeptidyl peptidase 4 |
|  | MOL000422 | kaempferol | Trypsin-1 |
|  | MOL000422 | kaempferol | Progesterone receptor |
|  | MOL000422 | kaempferol | Prothrombin |
|  | MOL000422 | kaempferol | Muscarinic acetylcholine receptor M1 |
|  | MOL000422 | kaempferol | Nitric-oxide synthase, endothelial |
|  | MOL000422 | kaempferol | Gamma-aminobutyric-acid receptor subunit alpha-2 |
|  | MOL000422 | kaempferol | Acetylcholinesterase |
|  | MOL000422 | kaempferol | Sodium-dependent noradrenaline transporter |
|  | MOL000422 | kaempferol | Muscarinic acetylcholine receptor M2 |
|  | MOL000422 | kaempferol | Alpha-1B adrenergic receptor |
|  | MOL000422 | kaempferol | Gamma-aminobutyric-acid receptor subunit alpha-1 |
|  | MOL000422 | kaempferol | DNA topoisomerase 2-alpha |
|  | MOL000422 | kaempferol | Coagulation factor VII |
|  | MOL000422 | kaempferol | Calmodulin |
|  | MOL000422 | kaempferol | Transcription factor p65 |
|  | MOL000422 | kaempferol | Inhibitor of nuclear factor kappa-B kinase subunit beta |
|  | MOL000422 | kaempferol | RAC-alpha serine/threonine-protein kinase |
|  | MOL000422 | kaempferol | Apoptosis regulator Bcl-2 |
|  | MOL000422 | kaempferol | Apoptosis regulator BAX |
|  | MOL000422 | kaempferol | Tumor necrosis factor |
|  | MOL000422 | kaempferol | Transcription factor AP-1 |
|  | MOL000422 | kaempferol | Activator of 90 kDa heat shock protein ATPase homolog 1 |
|  | MOL000422 | kaempferol | Caspase-3 |
|  | MOL000422 | kaempferol | Mitogen-activated protein kinase 8 |
|  | MOL000422 | kaempferol | Xanthine dehydrogenase/oxidase |
|  | MOL000422 | kaempferol | Interstitial collagenase |
|  | MOL000422 | kaempferol | Signal transducer and activator of transcription 1-alpha/beta |
|  | MOL000422 | kaempferol | Cell division control protein 2 homolog |
|  | MOL000422 | kaempferol | Peroxisome proliferator-activated receptor gamma |
|  | MOL000422 | kaempferol | Heme oxygenase 1 |
|  | MOL000422 | kaempferol | Cytochrome P450 3A4 |
|  | MOL000422 | kaempferol | Cytochrome P450 1A1 |
|  | MOL000422 | kaempferol | Intercellular adhesion molecule 1 |
|  | MOL000422 | kaempferol | E-selectin |
|  | MOL000422 | kaempferol | Vascular cell adhesion protein 1 |
|  | MOL000422 | kaempferol | Nuclear receptor subfamily 1 group I member 2 |
|  | MOL000422 | kaempferol | Cytochrome P450 1B1 |
|  | MOL000422 | kaempferol | Arachidonate 5-lipoxygenase |
|  | MOL000422 | kaempferol | Hyaluronan synthase 2 |
|  | MOL000422 | kaempferol | Aryl hydrocarbon receptor |
|  | MOL000422 | kaempferol | 26S proteasome non-ATPase regulatory subunit 3 |
|  | MOL000422 | kaempferol | Solute carrier family 2, facilitated glucose transporter member 4 |
|  | MOL000422 | kaempferol | Nuclear receptor subfamily 1 group I member 3 |
|  | MOL000422 | kaempferol | Insulin receptor |
|  | MOL000422 | kaempferol | Type I iodothyronine deiodinase |
|  | MOL000422 | kaempferol | Serine/threonine-protein phosphatase 2B catalytic subunit alpha isoform |
|  | MOL000422 | kaempferol | Peroxidase C1A |
|  | MOL000422 | kaempferol | Glutathione S-transferase Mu 1 |
|  | MOL000422 | kaempferol | Glutathione S-transferase Mu 2 |
|  | MOL000422 | kaempferol | Aldo-keto reductase family 1 member C3 |
|  | MOL000422 | kaempferol | Antileukoproteinase |
|  | MOL004598 | 3,5,6,7-tetramethoxy-2-(3,4,5-trimethoxyphenyl)chromone | Prothrombin |
|  | MOL004598 | 3,5,6,7-tetramethoxy-2-(3,4,5-trimethoxyphenyl)chromone | Estrogen receptor |
|  | MOL004598 | 3,5,6,7-tetramethoxy-2-(3,4,5-trimethoxyphenyl)chromone | Androgen receptor |
|  | MOL004598 | 3,5,6,7-tetramethoxy-2-(3,4,5-trimethoxyphenyl)chromone | Coagulation factor X |
|  | MOL004598 | 3,5,6,7-tetramethoxy-2-(3,4,5-trimethoxyphenyl)chromone | Prostaglandin G/H synthase 2 |
|  | MOL004598 | 3,5,6,7-tetramethoxy-2-(3,4,5-trimethoxyphenyl)chromone | Coagulation factor VII |
|  | MOL004598 | 3,5,6,7-tetramethoxy-2-(3,4,5-trimethoxyphenyl)chromone | Acetylcholinesterase |
|  | MOL004598 | 3,5,6,7-tetramethoxy-2-(3,4,5-trimethoxyphenyl)chromone | DNA topoisomerase 2-alpha |
|  | MOL004598 | 3,5,6,7-tetramethoxy-2-(3,4,5-trimethoxyphenyl)chromone | Estrogen receptor beta |
|  | MOL004598 | 3,5,6,7-tetramethoxy-2-(3,4,5-trimethoxyphenyl)chromone | Trypsin-1 |
|  | MOL004598 | 3,5,6,7-tetramethoxy-2-(3,4,5-trimethoxyphenyl)chromone | Nuclear receptor coactivator 2 |
|  | MOL004598 | 3,5,6,7-tetramethoxy-2-(3,4,5-trimethoxyphenyl)chromone | Calmodulin |
|  | MOL004609 | Areapillin | Nitric oxide synthase, inducible |
|  | MOL004609 | Areapillin | Prothrombin |
|  | MOL004609 | Areapillin | Androgen receptor |
|  | MOL004609 | Areapillin | Sodium channel protein type 5 subunit alpha |
|  | MOL004609 | Areapillin | Coagulation factor X |
|  | MOL004609 | Areapillin | Prostaglandin G/H synthase 2 |
|  | MOL004609 | Areapillin | Coagulation factor VII |
|  | MOL004609 | Areapillin | Tyrosine-protein phosphatase non-receptor type 1 |
|  | MOL004609 | Areapillin | DNA topoisomerase 2-alpha |
|  | MOL004609 | Areapillin | Estrogen receptor beta |
|  | MOL004609 | Areapillin | Dipeptidyl peptidase 4 |
|  | MOL004609 | Areapillin | Heat shock protein HSP 90-alpha |
|  | MOL004609 | Areapillin | Ig gamma-1 chain C region |
|  | MOL004609 | Areapillin | Trypsin-1 |
|  | MOL004609 | Areapillin | Nuclear receptor coactivator 2 |
|  | MOL004609 | Areapillin | Nuclear receptor coactivator 1 |
|  | MOL004609 | Areapillin | Calmodulin |
|  | MOL013187 | Cubebin | Prostaglandin G/H synthase 1 |
|  | MOL013187 | Cubebin | Coagulation factor X |
|  | MOL013187 | Cubebin | Prostaglandin G/H synthase 2 |
|  | MOL013187 | Cubebin | Beta-2 adrenergic receptor |
|  | MOL013187 | Cubebin | Heat shock protein HSP 90-alpha |
|  | MOL004624 | Longikaurin A | Muscarinic acetylcholine receptor M1 |
|  | MOL004624 | Longikaurin A | Gamma-aminobutyric-acid receptor subunit alpha-2 |
|  | MOL004624 | Longikaurin A | Gamma-aminobutyric-acid receptor subunit alpha-3 |
|  | MOL004624 | Longikaurin A | Muscarinic acetylcholine receptor M2 |
|  | MOL004624 | Longikaurin A | Trypsin-1 |
|  | MOL004624 | Longikaurin A | Gamma-aminobutyric-acid receptor subunit alpha-6 |
|  | MOL004653 | (+)-Anomalin | Prothrombin |
|  | MOL004653 | (+)-Anomalin | Potassium voltage-gated channel subfamily H member 2 |
|  | MOL004653 | (+)-Anomalin | Coagulation factor X |
|  | MOL004653 | (+)-Anomalin | Prostaglandin G/H synthase 2 |
|  | MOL004653 | (+)-Anomalin | DNA topoisomerase 2-alpha |
|  | MOL004653 | (+)-Anomalin | Dipeptidyl peptidase 4 |
|  | MOL004718 | Pseudo-ephedrine | Progesterone receptor |
|  | MOL004718 | Pseudo-ephedrine | Mineralocorticoid receptor |
|  | MOL004718 | Pseudo-ephedrine | Nuclear receptor coactivator 2 |
|  | MOL000490 | petunidin | Nitric oxide synthase, inducible |
|  | MOL000490 | petunidin | Prostaglandin G/H synthase 1 |
|  | MOL000490 | petunidin | Prostaglandin G/H synthase 2 |
|  | MOL000490 | petunidin | Estrogen receptor beta |
|  | MOL000490 | petunidin | Mitogen-activated protein kinase 14 |
|  | MOL000490 | petunidin | Glycogen synthase kinase-3 beta |
|  | MOL000490 | petunidin | Heat shock protein HSP 90-alpha |
|  | MOL000490 | petunidin | Nuclear receptor coactivator 2 |
|  | MOL000098 | quercetin | Prostaglandin G/H synthase 1 |
|  | MOL000098 | quercetin | Androgen receptor |
|  | MOL000098 | quercetin | Peroxisome proliferator-activated receptor gamma |
|  | MOL000098 | quercetin | Prostaglandin G/H synthase 2 |
|  | MOL000098 | quercetin | Heat shock protein HSP 90-alpha |
|  | MOL000098 | quercetin | Phosphatidylinositol-4,5-bisphosphate 3-kinase catalytic subunit gamma isoform |
|  | MOL000098 | quercetin | Nuclear receptor coactivator 2 |
|  | MOL000098 | quercetin | Dipeptidyl peptidase 4 |
|  | MOL000098 | quercetin | Aldose reductase |
|  | MOL000098 | quercetin | Trypsin-1 |
|  | MOL000098 | quercetin | DNA topoisomerase 2-alpha |
|  | MOL000098 | quercetin | Prothrombin |
|  | MOL000098 | quercetin | Potassium voltage-gated channel subfamily H member 2 |
|  | MOL000098 | quercetin | Sodium channel protein type 5 subunit alpha |
|  | MOL000098 | quercetin | Coagulation factor X |
|  | MOL000098 | quercetin | Beta-2 adrenergic receptor |
|  | MOL000098 | quercetin | Stromelysin-1 |
|  | MOL000098 | quercetin | cAMP-dependent protein kinase catalytic subunit alpha |
|  | MOL000098 | quercetin | Coagulation factor VII |
|  | MOL000098 | quercetin | Nitric-oxide synthase, endothelial |
|  | MOL000098 | quercetin | Retinoic acid receptor RXR-alpha |
|  | MOL000098 | quercetin | Acetylcholinesterase |
|  | MOL000098 | quercetin | Gamma-aminobutyric-acid receptor subunit alpha-1 |
|  | MOL000098 | quercetin | Amine oxidase [flavin-containing] B |
|  | MOL000098 | quercetin | Transcription factor p65 |
|  | MOL000098 | quercetin | Epidermal growth factor receptor |
|  | MOL000098 | quercetin | RAC-alpha serine/threonine-protein kinase |
|  | MOL000098 | quercetin |  |
|  | MOL000098 | quercetin | G1/S-specific cyclin-D1 |
|  | MOL000098 | quercetin | Apoptosis regulator Bcl-2 |
|  | MOL000098 | quercetin | Bcl-2-like protein 1 |
|  | MOL000098 | quercetin | Proto-oncogene c-Fos |
|  | MOL000098 | quercetin | Cyclin-dependent kinase inhibitor 1 |
|  | MOL000098 | quercetin | Eukaryotic translation initiation factor 6 |
|  | MOL000098 | quercetin | Apoptosis regulator BAX |
|  | MOL000098 | quercetin | Caspase-9 |
|  | MOL000098 | quercetin | Urokinase-type plasminogen activator |
|  | MOL000098 | quercetin | 72 kDa type IV collagenase |
|  | MOL000098 | quercetin | Matrix metalloproteinase-9 |
|  | MOL000098 | quercetin | Mitogen-activated protein kinase 1 |
|  | MOL000098 | quercetin | Interleukin-10 |
|  | MOL000098 | quercetin | Retinoblastoma-associated protein |
|  | MOL000098 | quercetin | Tumor necrosis factor |
|  | MOL000098 | quercetin | Transcription factor AP-1 |
|  | MOL000098 | quercetin | Interleukin-6 |
|  | MOL000098 | quercetin | Cyclin-dependent kinase inhibitor 2A, isoforms 1/2/3 |
|  | MOL000098 | quercetin | Activator of 90 kDa heat shock protein ATPase homolog 1 |
|  | MOL000098 | quercetin | Caspase-3 |
|  | MOL000098 | quercetin | Cellular tumor antigen p53 |
|  | MOL000098 | quercetin | ETS domain-containing protein Elk-1 |
|  | MOL000098 | quercetin | NF-kappa-B inhibitor alpha |
|  | MOL000098 | quercetin | Ornithine decarboxylase |
|  | MOL000098 | quercetin | Xanthine dehydrogenase/oxidase |
|  | MOL000098 | quercetin | Caspase-8 |
|  | MOL000098 | quercetin | DNA topoisomerase 1 |
|  | MOL000098 | quercetin | RAF proto-oncogene serine/threonine-protein kinase |
|  | MOL000098 | quercetin | Superoxide dismutase [Cu-Zn] |
|  | MOL000098 | quercetin | Protein kinase C alpha type |
|  | MOL000098 | quercetin | Interstitial collagenase |
|  | MOL000098 | quercetin | Hypoxia-inducible factor 1-alpha |
|  | MOL000098 | quercetin | Signal transducer and activator of transcription 1-alpha/beta |
|  | MOL000098 | quercetin | Protein CBFA2T1 |
|  | MOL000098 | quercetin | Probable E3 ubiquitin-protein ligase HERC5 |
|  | MOL000098 | quercetin | Cell division control protein 2 homolog |
|  | MOL000098 | quercetin | 78 kDa glucose-regulated protein |
|  | MOL000098 | quercetin | Receptor tyrosine-protein kinase erbB-2 |
|  | MOL000098 | quercetin | Peroxisome proliferator-activated receptor gamma |
|  | MOL000098 | quercetin | Acetyl-CoA carboxylase 1 |
|  | MOL000098 | quercetin | Heme oxygenase 1 |
|  | MOL000098 | quercetin | Cytochrome P450 3A4 |
|  | MOL000098 | quercetin | Caveolin-1 |
|  | MOL000098 | quercetin | Myc proto-oncogene protein |
|  | MOL000098 | quercetin | Tissue factor |
|  | MOL000098 | quercetin | Gap junction alpha-1 protein |
|  | MOL000098 | quercetin | Cytochrome P450 1A1 |
|  | MOL000098 | quercetin | Intercellular adhesion molecule 1 |
|  | MOL000098 | quercetin | Interleukin-1 beta |
|  | MOL000098 | quercetin | Small inducible cytokine A2 |
|  | MOL000098 | quercetin | E-selectin |
|  | MOL000098 | quercetin | Vascular cell adhesion protein 1 |
|  | MOL000098 | quercetin | Prostaglandin E2 receptor, EP3 subtype |
|  | MOL000098 | quercetin | Interleukin-8 |
|  | MOL000098 | quercetin | Protein kinase C beta type |
|  | MOL000098 | quercetin | Baculoviral IAP repeat-containing protein 5 |
|  | MOL000098 | quercetin | Dual oxidase 2 |
|  | MOL000098 | quercetin | Nitric oxide synthase, endothelial |
|  | MOL000098 | quercetin | Heat shock protein beta-1 |
|  | MOL000098 | quercetin | Transforming growth factor beta-1 |
|  | MOL000098 | quercetin | Maltase-glucoamylase, intestinal |
|  | MOL000098 | quercetin | Interleukin-2 |
|  | MOL000098 | quercetin | Nuclear receptor subfamily 1 group I member 2 |
|  | MOL000098 | quercetin | Cytochrome P450 1B1 |
|  | MOL000098 | quercetin | G2/mitotic-specific cyclin-B1 |
|  | MOL000098 | quercetin | Tissue-type plasminogen activator |
|  | MOL000098 | quercetin | Thrombomodulin |
|  | MOL000098 | quercetin | Plasminogen activator inhibitor 1 |
|  | MOL000098 | quercetin | Interferon gamma |
|  | MOL000098 | quercetin | Arachidonate 5-lipoxygenase |
|  | MOL000098 | quercetin | Phosphatidylinositol-3,4,5-trisphosphate 3-phosphatase and dual-specificity protein phosphatase PTEN |
|  | MOL000098 | quercetin | Interleukin-1 alpha |
|  | MOL000098 | quercetin | Myeloperoxidase |
|  | MOL000098 | quercetin | DNA topoisomerase 2-alpha |
|  | MOL000098 | quercetin | Neutrophil cytosol factor 1 |
|  | MOL000098 | quercetin | ATP-binding cassette sub-family G member 2 |
|  | MOL000098 | quercetin | Hyaluronan synthase 2 |
|  | MOL000098 | quercetin | Nuclear factor erythroid 2-related factor 2 |
|  | MOL000098 | quercetin | NAD(P)H dehydrogenase [quinone] 1 |
|  | MOL000098 | quercetin | Poly [ADP-ribose] polymerase 1 |
|  | MOL000098 | quercetin | Aryl hydrocarbon receptor |
|  | MOL000098 | quercetin | 26S proteasome non-ATPase regulatory subunit 3 |
|  | MOL000098 | quercetin | Solute carrier family 2, facilitated glucose transporter member 4 |
|  | MOL000098 | quercetin | Collagen alpha-1(III) chain |
|  | MOL000098 | quercetin | DNA gyrase subunit B |
|  | MOL000098 | quercetin | C-X-C motif chemokine 11 |
|  | MOL000098 | quercetin | C-X-C motif chemokine 2 |
|  | MOL000098 | quercetin | DDB1- and CUL4-associated factor 5 |
|  | MOL000098 | quercetin | Nuclear receptor subfamily 1 group I member 3 |
|  | MOL000098 | quercetin | Serine/threonine-protein kinase Chk2 |
|  | MOL000098 | quercetin | Insulin receptor |
|  | MOL000098 | quercetin | Claudin-4 |
|  | MOL000098 | quercetin | Peroxisome proliferator-activated receptor alpha |
|  | MOL000098 | quercetin | Peroxisome proliferator-activated receptor delta |
|  | MOL000098 | quercetin | Heat shock factor protein 1 |
|  | MOL000098 | quercetin | C-reactive protein |
|  | MOL000098 | quercetin | C-X-C motif chemokine 10 |
|  | MOL000098 | quercetin | Inhibitor of nuclear factor kappa-B kinase subunit alpha |
|  | MOL000098 | quercetin | Osteopontin |
|  | MOL000098 | quercetin | Runt-related transcription factor 2 |
|  | MOL000098 | quercetin | Ras association domain-containing protein 1 |
|  | MOL000098 | quercetin | Transcription factor E2F1 |
|  | MOL000098 | quercetin | Transcription factor E2F2 |
|  | MOL000098 | quercetin | Prostatic acid phosphatase |
|  | MOL000098 | quercetin | Cathepsin D |
|  | MOL000098 | quercetin | Insulin-like growth factor-binding protein 3 |
|  | MOL000098 | quercetin | Insulin-like growth factor II |
|  | MOL000098 | quercetin | CD40 ligand |
|  | MOL000098 | quercetin | Interferon regulatory factor 1 |
|  | MOL000098 | quercetin | Receptor tyrosine-protein kinase erbB-3 |
|  | MOL000098 | quercetin | Serum paraoxonase/arylesterase 1 |
|  | MOL000098 | quercetin | Type I iodothyronine deiodinase |
|  | MOL000098 | quercetin | Procollagen C-endopeptidase enhancer 1 |
|  | MOL000098 | quercetin | Puromycin-sensitive aminopeptidase |
|  | MOL000098 | quercetin | Hexokinase-2 |
|  | MOL000098 | quercetin | Homeobox protein Nkx-3.1 |
|  | MOL000098 | quercetin | Ras GTPase-activating protein 1 |
|  | MOL000098 | quercetin | Peroxidase C1A |
|  | MOL000098 | quercetin | Glutathione S-transferase Mu 1 |
|  | MOL000098 | quercetin | Glutathione S-transferase Mu 2 |
| Cannabis sativa L. [Cannabaceae, cannabis fructus] | Mol ID | Molecule Name | Target Name |
|  | MOL001439 | arachidonic acid | Prostaglandin G/H synthase 1 |
|  | MOL001439 | arachidonic acid | Prostaglandin G/H synthase 2 |
|  | MOL001439 | arachidonic acid | Retinoic acid receptor RXR-alpha |
|  | MOL001439 | arachidonic acid | Transient receptor potential cation channel subfamily V member 1 |
|  | MOL001439 | arachidonic acid | Retinoic acid receptor RXR-gamma |
|  | MOL001439 | arachidonic acid | Sodium-dependent noradrenaline transporter |
|  | MOL001439 | arachidonic acid | Transcription factor p65 |
|  | MOL001439 | arachidonic acid | G1/S-specific cyclin-D1 |
|  | MOL001439 | arachidonic acid | Mitogen-activated protein kinase 1 |
|  | MOL001439 | arachidonic acid |  |
|  | MOL001439 | arachidonic acid | Cell division protein kinase 4 |
|  | MOL001439 | arachidonic acid | Caspase-3 |
|  | MOL001439 | arachidonic acid | Probable E3 ubiquitin-protein ligase HERC5 |
|  | MOL001439 | arachidonic acid | Peroxisome proliferator-activated receptor gamma |
|  | MOL001439 | arachidonic acid | Glucose-6-phosphate 1-dehydrogenase |
|  | MOL001439 | arachidonic acid | Protein kinase C beta type |
|  | MOL001439 | arachidonic acid | Platelet endothelial cell adhesion molecule |
|  | MOL001439 | arachidonic acid | Nitric oxide synthase, endothelial |
|  | MOL001439 | arachidonic acid | Arachidonate 5-lipoxygenase |
|  | MOL001439 | arachidonic acid | Cytosolic phospholipase A2 |
|  | MOL001439 | arachidonic acid | Phosphatidylinositol-3,4,5-trisphosphate 3-phosphatase and dual-specificity protein phosphatase PTEN |
|  | MOL001439 | arachidonic acid | P-selectin |
|  | MOL001439 | arachidonic acid | Prostaglandin E synthase |
|  | MOL001439 | arachidonic acid | Beta-galactosidase |
|  | MOL001439 | arachidonic acid | Aldehyde dehydrogenase, mitochondrial |
|  | MOL001439 | arachidonic acid | ATP-binding cassette sub-family A member 1 |
|  | MOL001439 | arachidonic acid | Mitochondrial uncoupling protein 2 |
|  | MOL001439 | arachidonic acid | Complement C1r subcomponent |
|  | MOL001439 | arachidonic acid | Cholesteryl ester transfer protein |
|  | MOL001439 | arachidonic acid | Multidrug resistance-associated protein 4 |
|  | MOL001439 | arachidonic acid | Potassium channel subfamily K member 10 |
|  | MOL001439 | arachidonic acid | Tumor necrosis factor receptor superfamily member 1B |
|  | MOL001439 | arachidonic acid | Prostaglandin E synthase 2 |
|  | MOL001439 | arachidonic acid | Potassium channel subfamily K member 2 |
|  | MOL001439 | arachidonic acid | Collagen alpha-2(I) chain |
|  | MOL000359 | sitosterol | Progesterone receptor |
|  | MOL000359 | sitosterol | Nuclear receptor coactivator 2 |
|  | MOL000359 | sitosterol | Mineralocorticoid receptor |
|  | MOL000449 | Stigmasterol | Progesterone receptor |
|  | MOL000449 | Stigmasterol | Mineralocorticoid receptor |
|  | MOL000449 | Stigmasterol | Nuclear receptor coactivator 2 |
|  | MOL000449 | Stigmasterol | Ig gamma-1 chain C region |
|  | MOL000449 | Stigmasterol | Retinoic acid receptor RXR-alpha |
|  | MOL000449 | Stigmasterol | Nuclear receptor coactivator 1 |
|  | MOL000449 | Stigmasterol | Prostaglandin G/H synthase 1 |
|  | MOL000449 | Stigmasterol | Prostaglandin G/H synthase 2 |
|  | MOL000449 | Stigmasterol | Alpha-2A adrenergic receptor |
|  | MOL000449 | Stigmasterol | Sodium-dependent noradrenaline transporter |
|  | MOL000449 | Stigmasterol | Sodium-dependent dopamine transporter |
|  | MOL000449 | Stigmasterol | Beta-2 adrenergic receptor |
|  | MOL000449 | Stigmasterol | Aldose reductase |
|  | MOL000449 | Stigmasterol | Urokinase-type plasminogen activator |
|  | MOL000449 | Stigmasterol | Leukotriene A-4 hydrolase |
|  | MOL000449 | Stigmasterol | Amine oxidase [flavin-containing] B |
|  | MOL000449 | Stigmasterol | Amine oxidase [flavin-containing] A |
|  | MOL000449 | Stigmasterol | cAMP-dependent protein kinase catalytic subunit alpha |
|  | MOL000449 | Stigmasterol | Chymotrypsinogen B |
|  | MOL000449 | Stigmasterol | Muscarinic acetylcholine receptor M3 |
|  | MOL000449 | Stigmasterol | Muscarinic acetylcholine receptor M1 |
|  | MOL000449 | Stigmasterol | Beta-1 adrenergic receptor |
|  | MOL000449 | Stigmasterol | Sodium channel protein type 5 subunit alpha |
|  | MOL000449 | Stigmasterol | 5-hydroxytryptamine 2A receptor |
|  | MOL000449 | Stigmasterol | Alpha-1A adrenergic receptor |
|  | MOL000449 | Stigmasterol | Gamma-aminobutyric-acid receptor subunit alpha-3 |
|  | MOL000449 | Stigmasterol | Muscarinic acetylcholine receptor M2 |
|  | MOL000449 | Stigmasterol | Alpha-1B adrenergic receptor |
|  | MOL000449 | Stigmasterol | Gamma-aminobutyric-acid receptor subunit alpha-1 |
|  | MOL000449 | Stigmasterol | Neuronal acetylcholine receptor subunit alpha-7 |
|  | MOL000483 | (Z)-3-(4-hydroxy-3-methoxy-phenyl)-N-[2-(4-hydroxyphenyl)ethyl]acrylamide | Prostaglandin G/H synthase 1 |
|  | MOL000483 | (Z)-3-(4-hydroxy-3-methoxy-phenyl)-N-[2-(4-hydroxyphenyl)ethyl]acrylamide | Prostaglandin G/H synthase 2 |
|  | MOL000483 | (Z)-3-(4-hydroxy-3-methoxy-phenyl)-N-[2-(4-hydroxyphenyl)ethyl]acrylamide | cGMP-inhibited 3',5'-cyclic phosphodiesterase A |
|  | MOL000483 | (Z)-3-(4-hydroxy-3-methoxy-phenyl)-N-[2-(4-hydroxyphenyl)ethyl]acrylamide | Alpha-1B adrenergic receptor |
|  | MOL000483 | (Z)-3-(4-hydroxy-3-methoxy-phenyl)-N-[2-(4-hydroxyphenyl)ethyl]acrylamide | Beta-2 adrenergic receptor |
|  | MOL000483 | (Z)-3-(4-hydroxy-3-methoxy-phenyl)-N-[2-(4-hydroxyphenyl)ethyl]acrylamide | Heat shock protein HSP 90-alpha |
|  | MOL000483 | (Z)-3-(4-hydroxy-3-methoxy-phenyl)-N-[2-(4-hydroxyphenyl)ethyl]acrylamide | Leukotriene A-4 hydrolase |
|  | MOL000483 | (Z)-3-(4-hydroxy-3-methoxy-phenyl)-N-[2-(4-hydroxyphenyl)ethyl]acrylamide | Calmodulin |
|  | MOL005030 | gondoic acid | Prostaglandin G/H synthase 1 |
|  | MOL005030 | gondoic acid | Nuclear receptor coactivator 2 |
|  | MOL000006 | luteolin | Prostaglandin G/H synthase 1 |
|  | MOL000006 | luteolin | Androgen receptor |
|  | MOL000006 | luteolin | Prostaglandin G/H synthase 2 |
|  | MOL000006 | luteolin | Heat shock protein HSP 90-alpha |
|  | MOL000006 | luteolin | Trypsin-1 |
|  | MOL000006 | luteolin | Nuclear receptor coactivator 2 |
|  | MOL000006 | luteolin | cAMP-dependent protein kinase catalytic subunit alpha |
|  | MOL000006 | luteolin | Dipeptidyl peptidase 4 |
|  | MOL000006 | luteolin | Phosphatidylinositol-4,5-bisphosphate 3-kinase catalytic subunit gamma isoform |
|  | MOL000006 | luteolin | Transcription factor p65 |
|  | MOL000006 | luteolin | Epidermal growth factor receptor |
|  | MOL000006 | luteolin | RAC-alpha serine/threonine-protein kinase |
|  | MOL000006 | luteolin |  |
|  | MOL000006 | luteolin | G1/S-specific cyclin-D1 |
|  | MOL000006 | luteolin | Bcl-2-like protein 1 |
|  | MOL000006 | luteolin | Cyclin-dependent kinase inhibitor 1 |
|  | MOL000006 | luteolin | Caspase-9 |
|  | MOL000006 | luteolin | 72 kDa type IV collagenase |
|  | MOL000006 | luteolin | Matrix metalloproteinase-9 |
|  | MOL000006 | luteolin | Mitogen-activated protein kinase 1 |
|  | MOL000006 | luteolin | Interleukin-10 |
|  | MOL000006 | luteolin | Retinoblastoma-associated protein |
|  | MOL000006 | luteolin | Cell division protein kinase 4 |
|  | MOL000006 | luteolin | Tumor necrosis factor |
|  | MOL000006 | luteolin | Transcription factor AP-1 |
|  | MOL000006 | luteolin | Interleukin-6 |
|  | MOL000006 | luteolin | Caspase-3 |
|  | MOL000006 | luteolin | Cellular tumor antigen p53 |
|  | MOL000006 | luteolin | NF-kappa-B inhibitor alpha |
|  | MOL000006 | luteolin | Xanthine dehydrogenase/oxidase |
|  | MOL000006 | luteolin | DNA topoisomerase 1 |
|  | MOL000006 | luteolin | E3 ubiquitin-protein ligase Mdm2 |
|  | MOL000006 | luteolin | Amyloid beta A4 protein |
|  | MOL000006 | luteolin | Interstitial collagenase |
|  | MOL000006 | luteolin | Proliferating cell nuclear antigen |
|  | MOL000006 | luteolin | Receptor tyrosine-protein kinase erbB-2 |
|  | MOL000006 | luteolin | Peroxisome proliferator-activated receptor gamma |
|  | MOL000006 | luteolin | Heme oxygenase 1 |
|  | MOL000006 | luteolin | Caspase-7 |
|  | MOL000006 | luteolin | Intercellular adhesion molecule 1 |
|  | MOL000006 | luteolin | Induced myeloid leukemia cell differentiation protein Mcl-1 |
|  | MOL000006 | luteolin | Baculoviral IAP repeat-containing protein 5 |
|  | MOL000006 | luteolin | Interleukin-2 |
|  | MOL000006 | luteolin | G2/mitotic-specific cyclin-B1 |
|  | MOL000006 | luteolin | Tyrosinase |
|  | MOL000006 | luteolin | Interferon gamma |
|  | MOL000006 | luteolin | Interleukin-4 |
|  | MOL000006 | luteolin | DNA topoisomerase 2-alpha |
|  | MOL000006 | luteolin | Baculoviral IAP repeat-containing protein 4 |
|  | MOL000006 | luteolin | Solute carrier family 2, facilitated glucose transporter member 4 |
|  | MOL000006 | luteolin | Insulin receptor |
|  | MOL000006 | luteolin | CD40 ligand |
|  | MOL000006 | luteolin | Prostaglandin E synthase |
|  | MOL000006 | luteolin | Kinetochore protein Nuf2 |
|  | MOL000006 | luteolin | Adenylate cyclase type 2 |
|  | MOL000006 | luteolin | Hepatocyte growth factor receptor |
|  | MOL001389 | n-heptanal | Gamma-aminobutyric-acid receptor subunit alpha-2 |
|  | MOL001389 | n-heptanal | Acetylcholinesterase |
|  | MOL001389 | n-heptanal | Gamma-aminobutyric-acid receptor subunit alpha-1 |
|  | MOL001389 | n-heptanal | Cytochrome P450-cam |
|  | MOL001406 | crocetin | Cytochrome P450-cam |
|  | MOL001406 | crocetin | Muscarinic acetylcholine receptor M3 |
|  | MOL001406 | crocetin | Muscarinic acetylcholine receptor M1 |
|  | MOL001406 | crocetin | Gamma-aminobutyric-acid receptor subunit alpha-2 |
|  | MOL001406 | crocetin | Gamma-aminobutyric-acid receptor subunit alpha-5 |
|  | MOL001406 | crocetin | Alpha-1A adrenergic receptor |
|  | MOL001406 | crocetin | Gamma-aminobutyric-acid receptor subunit alpha-3 |
|  | MOL001406 | crocetin | Muscarinic acetylcholine receptor M2 |
|  | MOL001406 | crocetin | Alpha-1B adrenergic receptor |
|  | MOL001406 | crocetin | Gamma-aminobutyric-acid receptor subunit alpha-1 |
|  | MOL001406 | crocetin | Ig gamma-1 chain C region |
|  | MOL001406 | crocetin | Prostaglandin G/H synthase 2 |
|  | MOL001406 | crocetin | Nuclear receptor coactivator 2 |
|  | MOL001406 | crocetin | Vascular cell adhesion protein 1 |
|  | MOL000354 | isorhamnetin | Nitric oxide synthase, inducible |
|  | MOL000354 | isorhamnetin | Prostaglandin G/H synthase 1 |
|  | MOL000354 | isorhamnetin | Estrogen receptor |
|  | MOL000354 | isorhamnetin | Androgen receptor |
|  | MOL000354 | isorhamnetin | Peroxisome proliferator-activated receptor gamma |
|  | MOL000354 | isorhamnetin | Prostaglandin G/H synthase 2 |
|  | MOL000354 | isorhamnetin | Tyrosine-protein phosphatase non-receptor type 1 |
|  | MOL000354 | isorhamnetin | Estrogen receptor beta |
|  | MOL000354 | isorhamnetin | Dipeptidyl peptidase 4 |
|  | MOL000354 | isorhamnetin | Mitogen-activated protein kinase 14 |
|  | MOL000354 | isorhamnetin | Glycogen synthase kinase-3 beta |
|  | MOL000354 | isorhamnetin | Heat shock protein HSP 90-alpha |
|  | MOL000354 | isorhamnetin | Cell division protein kinase 2 |
|  | MOL000354 | isorhamnetin | Phosphatidylinositol-4,5-bisphosphate 3-kinase catalytic subunit gamma isoform |
|  | MOL000354 | isorhamnetin | cAMP-dependent protein kinase catalytic subunit alpha |
|  | MOL000354 | isorhamnetin | Trypsin-1 |
|  | MOL000354 | isorhamnetin | Proto-oncogene serine/threonine-protein kinase Pim-1 |
|  | MOL000354 | isorhamnetin | Cyclin-A2 |
|  | MOL000354 | isorhamnetin | Nuclear receptor coactivator 2 |
|  | MOL000354 | isorhamnetin | Calmodulin |
|  | MOL000354 | isorhamnetin | Glycogen phosphorylase, muscle form |
|  | MOL000354 | isorhamnetin | Peroxisome proliferator-activated receptor delta |
|  | MOL000354 | isorhamnetin | Serine/threonine-protein kinase Chk1 |
|  | MOL000354 | isorhamnetin | Aldose reductase |
|  | MOL000354 | isorhamnetin | Nuclear receptor coactivator 1 |
|  | MOL000354 | isorhamnetin | Coagulation factor VII |
|  | MOL000354 | isorhamnetin | Prothrombin |
|  | MOL000354 | isorhamnetin | Nitric-oxide synthase, endothelial |
|  | MOL000354 | isorhamnetin | Acetylcholinesterase |
|  | MOL000354 | isorhamnetin | Gamma-aminobutyric-acid receptor subunit alpha-1 |
|  | MOL000354 | isorhamnetin | Amine oxidase [flavin-containing] B |
|  | MOL000354 | isorhamnetin | Glutamate receptor 2 |
|  | MOL000354 | isorhamnetin | Cytochrome P450-cam |
|  | MOL000354 | isorhamnetin | Transcription factor p65 |
|  | MOL000354 | isorhamnetin | Xanthine dehydrogenase/oxidase |
|  | MOL000354 | isorhamnetin | Neutrophil cytosol factor 1 |
|  | MOL000354 | isorhamnetin | Oxidized low-density lipoprotein receptor 1 |
|  | MOL000422 | kaempferol | Nitric oxide synthase, inducible |
|  | MOL000422 | kaempferol | Prostaglandin G/H synthase 1 |
|  | MOL000422 | kaempferol | Androgen receptor |
|  | MOL000422 | kaempferol | Peroxisome proliferator-activated receptor gamma |
|  | MOL000422 | kaempferol | Prostaglandin G/H synthase 2 |
|  | MOL000422 | kaempferol | Heat shock protein HSP 90-alpha |
|  | MOL000422 | kaempferol | Phosphatidylinositol-4,5-bisphosphate 3-kinase catalytic subunit gamma isoform |
|  | MOL000422 | kaempferol | cAMP-dependent protein kinase catalytic subunit alpha |
|  | MOL000422 | kaempferol | Nuclear receptor coactivator 2 |
|  | MOL000422 | kaempferol | Dipeptidyl peptidase 4 |
|  | MOL000422 | kaempferol | Trypsin-1 |
|  | MOL000422 | kaempferol | Progesterone receptor |
|  | MOL000422 | kaempferol | Prothrombin |
|  | MOL000422 | kaempferol | Muscarinic acetylcholine receptor M1 |
|  | MOL000422 | kaempferol | Nitric-oxide synthase, endothelial |
|  | MOL000422 | kaempferol | Gamma-aminobutyric-acid receptor subunit alpha-2 |
|  | MOL000422 | kaempferol | Acetylcholinesterase |
|  | MOL000422 | kaempferol | Sodium-dependent noradrenaline transporter |
|  | MOL000422 | kaempferol | Muscarinic acetylcholine receptor M2 |
|  | MOL000422 | kaempferol | Alpha-1B adrenergic receptor |
|  | MOL000422 | kaempferol | Gamma-aminobutyric-acid receptor subunit alpha-1 |
|  | MOL000422 | kaempferol | DNA topoisomerase 2-alpha |
|  | MOL000422 | kaempferol | Coagulation factor VII |
|  | MOL000422 | kaempferol | Calmodulin |
|  | MOL000422 | kaempferol | Transcription factor p65 |
|  | MOL000422 | kaempferol | Inhibitor of nuclear factor kappa-B kinase subunit beta |
|  | MOL000422 | kaempferol | RAC-alpha serine/threonine-protein kinase |
|  | MOL000422 | kaempferol | Apoptosis regulator Bcl-2 |
|  | MOL000422 | kaempferol | Apoptosis regulator BAX |
|  | MOL000422 | kaempferol | Tumor necrosis factor |
|  | MOL000422 | kaempferol | Transcription factor AP-1 |
|  | MOL000422 | kaempferol | Activator of 90 kDa heat shock protein ATPase homolog 1 |
|  | MOL000422 | kaempferol | Caspase-3 |
|  | MOL000422 | kaempferol | Mitogen-activated protein kinase 8 |
|  | MOL000422 | kaempferol | Xanthine dehydrogenase/oxidase |
|  | MOL000422 | kaempferol | Interstitial collagenase |
|  | MOL000422 | kaempferol | Signal transducer and activator of transcription 1-alpha/beta |
|  | MOL000422 | kaempferol | Cell division control protein 2 homolog |
|  | MOL000422 | kaempferol | Peroxisome proliferator-activated receptor gamma |
|  | MOL000422 | kaempferol | Heme oxygenase 1 |
|  | MOL000422 | kaempferol | Cytochrome P450 3A4 |
|  | MOL000422 | kaempferol | Cytochrome P450 1A1 |
|  | MOL000422 | kaempferol | Intercellular adhesion molecule 1 |
|  | MOL000422 | kaempferol | E-selectin |
|  | MOL000422 | kaempferol | Vascular cell adhesion protein 1 |
|  | MOL000422 | kaempferol | Nuclear receptor subfamily 1 group I member 2 |
|  | MOL000422 | kaempferol | Cytochrome P450 1B1 |
|  | MOL000422 | kaempferol | Arachidonate 5-lipoxygenase |
|  | MOL000422 | kaempferol | Hyaluronan synthase 2 |
|  | MOL000422 | kaempferol | Aryl hydrocarbon receptor |
|  | MOL000422 | kaempferol | 26S proteasome non-ATPase regulatory subunit 3 |
|  | MOL000422 | kaempferol | Solute carrier family 2, facilitated glucose transporter member 4 |
|  | MOL000422 | kaempferol | Nuclear receptor subfamily 1 group I member 3 |
|  | MOL000422 | kaempferol | Insulin receptor |
|  | MOL000422 | kaempferol | Type I iodothyronine deiodinase |
|  | MOL000422 | kaempferol | Serine/threonine-protein phosphatase 2B catalytic subunit alpha isoform |
|  | MOL000422 | kaempferol | Peroxidase C1A |
|  | MOL000422 | kaempferol | Glutathione S-transferase Mu 1 |
|  | MOL000422 | kaempferol | Glutathione S-transferase Mu 2 |
|  | MOL000422 | kaempferol | Aldo-keto reductase family 1 member C3 |
|  | MOL000422 | kaempferol | Antileukoproteinase |
|  | MOL000098 | quercetin | Prostaglandin G/H synthase 1 |
|  | MOL000098 | quercetin | Androgen receptor |
|  | MOL000098 | quercetin | Peroxisome proliferator-activated receptor gamma |
|  | MOL000098 | quercetin | Prostaglandin G/H synthase 2 |
|  | MOL000098 | quercetin | Heat shock protein HSP 90-alpha |
|  | MOL000098 | quercetin | Phosphatidylinositol-4,5-bisphosphate 3-kinase catalytic subunit gamma isoform |
|  | MOL000098 | quercetin | Nuclear receptor coactivator 2 |
|  | MOL000098 | quercetin | Dipeptidyl peptidase 4 |
|  | MOL000098 | quercetin | Aldose reductase |
|  | MOL000098 | quercetin | Trypsin-1 |
|  | MOL000098 | quercetin | DNA topoisomerase 2-alpha |
|  | MOL000098 | quercetin | Prothrombin |
|  | MOL000098 | quercetin | Potassium voltage-gated channel subfamily H member 2 |
|  | MOL000098 | quercetin | Sodium channel protein type 5 subunit alpha |
|  | MOL000098 | quercetin | Coagulation factor X |
|  | MOL000098 | quercetin | Beta-2 adrenergic receptor |
|  | MOL000098 | quercetin | Stromelysin-1 |
|  | MOL000098 | quercetin | cAMP-dependent protein kinase catalytic subunit alpha |
|  | MOL000098 | quercetin | Coagulation factor VII |
|  | MOL000098 | quercetin | Nitric-oxide synthase, endothelial |
|  | MOL000098 | quercetin | Retinoic acid receptor RXR-alpha |
|  | MOL000098 | quercetin | Acetylcholinesterase |
|  | MOL000098 | quercetin | Gamma-aminobutyric-acid receptor subunit alpha-1 |
|  | MOL000098 | quercetin | Amine oxidase [flavin-containing] B |
|  | MOL000098 | quercetin | Transcription factor p65 |
|  | MOL000098 | quercetin | Epidermal growth factor receptor |
|  | MOL000098 | quercetin | RAC-alpha serine/threonine-protein kinase |
|  | MOL000098 | quercetin | G1/S-specific cyclin-D1 |
|  | MOL000098 | quercetin | Apoptosis regulator Bcl-2 |
|  | MOL000098 | quercetin | Bcl-2-like protein 1 |
|  | MOL000098 | quercetin | Proto-oncogene c-Fos |
|  | MOL000098 | quercetin | Cyclin-dependent kinase inhibitor 1 |
|  | MOL000098 | quercetin | Eukaryotic translation initiation factor 6 |
|  | MOL000098 | quercetin | Apoptosis regulator BAX |
|  | MOL000098 | quercetin | Caspase-9 |
|  | MOL000098 | quercetin | Urokinase-type plasminogen activator |
|  | MOL000098 | quercetin | 72 kDa type IV collagenase |
|  | MOL000098 | quercetin | Matrix metalloproteinase-9 |
|  | MOL000098 | quercetin | Mitogen-activated protein kinase 1 |
|  | MOL000098 | quercetin | Interleukin-10 |
|  | MOL000098 | quercetin | Retinoblastoma-associated protein |
|  | MOL000098 | quercetin | Tumor necrosis factor |
|  | MOL000098 | quercetin | Transcription factor AP-1 |
|  | MOL000098 | quercetin | Interleukin-6 |
|  | MOL000098 | quercetin | Cyclin-dependent kinase inhibitor 2A, isoforms 1/2/3 |
|  | MOL000098 | quercetin | Activator of 90 kDa heat shock protein ATPase homolog 1 |
|  | MOL000098 | quercetin | Caspase-3 |
|  | MOL000098 | quercetin | Cellular tumor antigen p53 |
|  | MOL000098 | quercetin | ETS domain-containing protein Elk-1 |
|  | MOL000098 | quercetin | NF-kappa-B inhibitor alpha |
|  | MOL000098 | quercetin | Ornithine decarboxylase |
|  | MOL000098 | quercetin | Xanthine dehydrogenase/oxidase |
|  | MOL000098 | quercetin | Caspase-8 |
|  | MOL000098 | quercetin | DNA topoisomerase 1 |
|  | MOL000098 | quercetin | RAF proto-oncogene serine/threonine-protein kinase |
|  | MOL000098 | quercetin | Superoxide dismutase [Cu-Zn] |
|  | MOL000098 | quercetin | Protein kinase C alpha type |
|  | MOL000098 | quercetin | Interstitial collagenase |
|  | MOL000098 | quercetin | Hypoxia-inducible factor 1-alpha |
|  | MOL000098 | quercetin | Signal transducer and activator of transcription 1-alpha/beta |
|  | MOL000098 | quercetin | Protein CBFA2T1 |
|  | MOL000098 | quercetin | Probable E3 ubiquitin-protein ligase HERC5 |
|  | MOL000098 | quercetin | Cell division control protein 2 homolog |
|  | MOL000098 | quercetin | 78 kDa glucose-regulated protein |
|  | MOL000098 | quercetin | Receptor tyrosine-protein kinase erbB-2 |
|  | MOL000098 | quercetin | Peroxisome proliferator-activated receptor gamma |
|  | MOL000098 | quercetin | Acetyl-CoA carboxylase 1 |
|  | MOL000098 | quercetin | Heme oxygenase 1 |
|  | MOL000098 | quercetin | Cytochrome P450 3A4 |
|  | MOL000098 | quercetin | Caveolin-1 |
|  | MOL000098 | quercetin | Myc proto-oncogene protein |
|  | MOL000098 | quercetin | Tissue factor |
|  | MOL000098 | quercetin | Gap junction alpha-1 protein |
|  | MOL000098 | quercetin | Cytochrome P450 1A1 |
|  | MOL000098 | quercetin | Intercellular adhesion molecule 1 |
|  | MOL000098 | quercetin | Interleukin-1 beta |
|  | MOL000098 | quercetin | Small inducible cytokine A2 |
|  | MOL000098 | quercetin | E-selectin |
|  | MOL000098 | quercetin | Vascular cell adhesion protein 1 |
|  | MOL000098 | quercetin | Prostaglandin E2 receptor, EP3 subtype |
|  | MOL000098 | quercetin | Interleukin-8 |
|  | MOL000098 | quercetin | Protein kinase C beta type |
|  | MOL000098 | quercetin | Baculoviral IAP repeat-containing protein 5 |
|  | MOL000098 | quercetin | Dual oxidase 2 |
|  | MOL000098 | quercetin | Nitric oxide synthase, endothelial |
|  | MOL000098 | quercetin | Heat shock protein beta-1 |
|  | MOL000098 | quercetin | Transforming growth factor beta-1 |
|  | MOL000098 | quercetin | Maltase-glucoamylase, intestinal |
|  | MOL000098 | quercetin | Interleukin-2 |
|  | MOL000098 | quercetin | Nuclear receptor subfamily 1 group I member 2 |
|  | MOL000098 | quercetin | Cytochrome P450 1B1 |
|  | MOL000098 | quercetin | G2/mitotic-specific cyclin-B1 |
|  | MOL000098 | quercetin | Tissue-type plasminogen activator |
|  | MOL000098 | quercetin | Thrombomodulin |
|  | MOL000098 | quercetin | Plasminogen activator inhibitor 1 |
|  | MOL000098 | quercetin | Interferon gamma |
|  | MOL000098 | quercetin | Arachidonate 5-lipoxygenase |
|  | MOL000098 | quercetin | Phosphatidylinositol-3,4,5-trisphosphate 3-phosphatase and dual-specificity protein phosphatase PTEN |
|  | MOL000098 | quercetin | Interleukin-1 alpha |
|  | MOL000098 | quercetin | Myeloperoxidase |
|  | MOL000098 | quercetin | DNA topoisomerase 2-alpha |
|  | MOL000098 | quercetin | Neutrophil cytosol factor 1 |
|  | MOL000098 | quercetin | ATP-binding cassette sub-family G member 2 |
|  | MOL000098 | quercetin | Hyaluronan synthase 2 |
|  | MOL000098 | quercetin | Nuclear factor erythroid 2-related factor 2 |
|  | MOL000098 | quercetin | NAD(P)H dehydrogenase [quinone] 1 |
|  | MOL000098 | quercetin | Poly [ADP-ribose] polymerase 1 |
|  | MOL000098 | quercetin | Aryl hydrocarbon receptor |
|  | MOL000098 | quercetin | 26S proteasome non-ATPase regulatory subunit 3 |
|  | MOL000098 | quercetin | Solute carrier family 2, facilitated glucose transporter member 4 |
|  | MOL000098 | quercetin | Collagen alpha-1(III) chain |
|  | MOL000098 | quercetin | DNA gyrase subunit B |
|  | MOL000098 | quercetin | C-X-C motif chemokine 11 |
|  | MOL000098 | quercetin | C-X-C motif chemokine 2 |
|  | MOL000098 | quercetin | DDB1- and CUL4-associated factor 5 |
|  | MOL000098 | quercetin | Nuclear receptor subfamily 1 group I member 3 |
|  | MOL000098 | quercetin | Serine/threonine-protein kinase Chk2 |
|  | MOL000098 | quercetin | Insulin receptor |
|  | MOL000098 | quercetin | Claudin-4 |
|  | MOL000098 | quercetin | Peroxisome proliferator-activated receptor alpha |
|  | MOL000098 | quercetin | Peroxisome proliferator-activated receptor delta |
|  | MOL000098 | quercetin | Heat shock factor protein 1 |
|  | MOL000098 | quercetin | C-reactive protein |
|  | MOL000098 | quercetin | C-X-C motif chemokine 10 |
|  | MOL000098 | quercetin | Inhibitor of nuclear factor kappa-B kinase subunit alpha |
|  | MOL000098 | quercetin | Osteopontin |
|  | MOL000098 | quercetin | Runt-related transcription factor 2 |
|  | MOL000098 | quercetin | Ras association domain-containing protein 1 |
|  | MOL000098 | quercetin | Transcription factor E2F1 |
|  | MOL000098 | quercetin | Transcription factor E2F2 |
|  | MOL000098 | quercetin | Prostatic acid phosphatase |
|  | MOL000098 | quercetin | Cathepsin D |
|  | MOL000098 | quercetin | Insulin-like growth factor-binding protein 3 |
|  | MOL000098 | quercetin | Insulin-like growth factor II |
|  | MOL000098 | quercetin | CD40 ligand |
|  | MOL000098 | quercetin | Interferon regulatory factor 1 |
|  | MOL000098 | quercetin | Receptor tyrosine-protein kinase erbB-3 |
|  | MOL000098 | quercetin | Serum paraoxonase/arylesterase 1 |
|  | MOL000098 | quercetin | Type I iodothyronine deiodinase |
|  | MOL000098 | quercetin | Procollagen C-endopeptidase enhancer 1 |
|  | MOL000098 | quercetin | Puromycin-sensitive aminopeptidase |
|  | MOL000098 | quercetin | Hexokinase-2 |
|  | MOL000098 | quercetin | Homeobox protein Nkx-3.1 |
|  | MOL000098 | quercetin | Ras GTPase-activating protein 1 |
|  | MOL000098 | quercetin | Peroxidase C1A |
|  | MOL000098 | quercetin | Glutathione S-transferase Mu 1 |
|  | MOL000098 | quercetin | Glutathione S-transferase Mu 2 |
